# Transplantation of Neural Precursors Derived from Induced Pluripotent Cells Preserve Perineuronal Nets and Stimulate Neural Plasticity in ALS Rats

**DOI:** 10.3390/ijms21249593

**Published:** 2020-12-16

**Authors:** Serhiy Forostyak, Oksana Forostyak, Jessica C. F. Kwok, Nataliya Romanyuk, Monika Rehorova, Jan Kriska, Govindan Dayanithi, Ruma Raha-Chowdhury, Pavla Jendelova, Miroslava Anderova, James W. Fawcett, Eva Sykova

**Affiliations:** 1 Institute of Experimental Medicine, Czech Academy of Sciences, Videnska 1083, 142 20 Prague, Czech Republic; oksana.forostyak@iem.cas.cz (O.F.); J.Kwok@leeds.ac.uk (J.C.F.K.); nataliya.romanyuk@iem.cas.cz (N.R.); monika.rehorova@lfmotol.cuni.cz (M.R.); jan.kriska@iem.cas.cz (J.K.); govindan.dayanithi@umontpellier.fr (G.D.); Pavla.jendelova@iem.cas.cz (P.J.); miroslava.anderova@iem.cas.cz (M.A.); sykovae@gmail.com (E.S.); 2 Second Faculty of Medicine, Charles University, V Úvalu 84, 150 06 Prague, Czech Republic; 3 John van Geest Centre for Brain Repair (BRC), Department of Clinical Neurosciences, University of Cambridge, Cambridge CB2 0PY, UK; ruma.rahachowdhury@gmail.com

**Keywords:** proteoglycans, plasticity, neurodegeneration, stem cells, iPS, ALS, motoneuron death, transplantation

## Abstract

A promising therapeutic strategy for amyotrophic lateral sclerosis (ALS) treatment is stem cell therapy. Neural progenitors derived from induced pluripotent cells (NP-iPS) might rescue or replace dying motoneurons (MNs). However, the mechanisms responsible for the beneficial effect are not fully understood. The aim here was to investigate the mechanism by studying the effect of intraspinally injected NP-iPS into asymptomatic and early symptomatic superoxide dismutase (SOD)1^G93A^ transgenic rats. Prior to transplantation, NP-iPS were characterized in vitro for their ability to differentiate into a neuronal phenotype. Motor functions were tested in all animals, and the tissue was analyzed by immunohistochemistry, qPCR, and Western blot. NP-iPS transplantation significantly preserved MNs, slowed disease progression, and extended the survival of all treated animals. The dysregulation of spinal chondroitin sulfate proteoglycans was observed in SOD1^G93A^ rats at the terminal stage. NP-iPS application led to normalized host genes expression (*versican, has-1, tenascin-R, ngf, igf-1, bdnf, bax, bcl-2, and casp-3*) and the protection of perineuronal nets around the preserved MNs. In the host spinal cord, transplanted cells remained as progenitors, many in contact with MNs, but they did not differentiate. The findings suggest that NP-iPS demonstrate neuroprotective properties by regulating local gene expression and regulate plasticity by modulating the central nervous system (CNS) extracellular matrix such as perineuronal nets (PNNs).

## 1. Introduction

Amyotrophic lateral sclerosis (ALS) is a complex, progressive neurodegenerative disorder, causing general muscle weakness, atrophy, and palsy, leading to eventual respiratory failure and death. The majority of ALS cases are sporadic (sALS), and only about 5–10% are associated with a large number of gene mutations and variants in more than 30 human chromosomal regions [1,2]. The most studied familial ALS (fALS) mutation is linked to the mutation in Zn/Cu superoxide dismutase (SOD1), which accounts for 20% of fALS cases [3]. Patients present a wide range of diverse clinical outcomes regarding disease onset, rate of progression, and survival [4]. Despite a number of hypotheses proposed to explain the disease-associated loss of motoneurons (MNs), the precise molecular mechanisms remain elusive, and no effective prophylactic or therapeutic treatments exist.

The breakdown of neuron–glia communication and a toxic gain of function are implicated in neuronal dysfunction and death in ALS [5,6]. The pathology of ALS also involves the surrounding glial cells, both astrocytes and microglia, and the glial cells from ALS model animals induce toxicity to MNs [7,8]. On the other hand, astrocytes together with various stem cell and progenitor types have demonstrated strong neuroprotective actions, and the transplantation of such cells into ALS models preserves MNs [9,10]. Induced pluripotent stem cells (iPSCs) and neural precursor cells derived from iPSC (NP-iPSCs) presented new perspectives for the modeling and treatment of neurodegenerative disorders. They are able to differentiate into neural subtypes including neurons, MNs, astrocytes, oligodendrocytes, and Schwann cells. It is possible to develop cell subtypes from ALS patients with known genotypes and phenotypes [11,12,13,14]. In general, NP-iPSCs can differentiate into neuronal and glial cells, and they have been shown to possess neuroprotective effects via the secretion of growth factors (paracrine function) and exosomes, modification of the glial scar, and stimulation of migration, differentiation, and even tissue regeneration after the administration [15,16,17,18]. These features resulted in an approved clinical trial to rescue the pigmented epithelium during severe age-related macular degeneration [19]. In addition, several groups were granted FDA approval for phase I clinical trials after demonstrating that neural progenitor administration can change the outcome of ALS [9,20].

To be effective in neuroprotection, implanted cells must remain in a protective state and must have some abilities to migrate to and stay in close proximity to MNs. A dialogue between host and transplanted cells will occur, which will affect the differentiation of implanted progenitors and modify the ALS disease process. Stem cells from different origin were utilized in experimental models of ALS [9,20,21,22,23,24]. They range from non-neural cells, such as mesenchymal stem cells (MSCs) to neural progenitors derived from embryonic stem cells or iPSC. Their application usually leads to the generation of a protective environment for degenerating neurons, tissue repair, and disease attenuation. In our previous studies, we used rat (rMSCs) or human (hMSCs), which were applied intraspinally and intravenously or intrathecally [21,22]. We have observed delay in the loss of motor functions and an increased overall survival of symptomatic ALS animals. Since we observed prolonged lifespan after intraspinal application of stem cells that are not neural origin (rMSCs) and thus do not differentiate into neural phenotypes, we decided to examine the effect of intraspinal application of neural origin stem cells. In this study, we have studied a well-established neural progenitor line, (NP-iPS) clone IMR90. These cells were derived from human embryonic lung fibroblasts transduced with lentivirus-mediated combination OCT4, SOX2, NANOG, and LIN28 [25]. In previous studies, these cells have been transplanted into ischemic brain and spinal cord lesions, where the cells differentiate into neuronal and glial phenotypes, restore functions, and protect the host tissue [17,25]. These cells are migratory, have neuronal differentiation ability, and can be neuroprotective. The neuroprotection caused by the transplants gives the opportunity to study changes in the extracellular matrix (ECM). Similarly, to our previous study, we assessed the effect of cell grafts on perineuronal nets (PNNs), which have recently been shown to be strongly protective against a range of toxic insults [26,27] and can be affected in neurodegenerative diseases. Earlier, we showed that the intrathecal application of hMSC to a certain extent preserved the structure of perineuronal nets (PNNs), which was accompanied by a delayed degeneration of MNs. Considering that the used animal model affects MNs and glia in the whole spinal cord (primarily lumbar), we hypothesized that the grafting of cells at the T10-T11 level is a perfect location for caudal and cranial migration within the spinal cord, and it shall provide a broader range of contact between the graft and host cells in order to facilitate regeneration. We also aimed to prevent the risk of damaging lumbar MNs for more accurate testing of the animals [22]. We also hypothesized that NP-iPS will more readily migrate along the spinal cord using the mentioned area for grafting the cells, and that NP-iPS might differentiate into glial or neuronal phenotypes that will interact with PNNs around the soma and proximal dendrites of inter- and motoneurons [28].

Therefore, the aim of our study was to assess the differentiation ability (in vitro and in vivo), survival, and neuroprotective effect of NP-iPS after intraspinal transplantation into asymptomatic and symptomatic SOD1^G93A^ transgenic rats.

## 2. Results

### 2.1. Differentiation of NP-iPS in Culture

**Figure 1:**
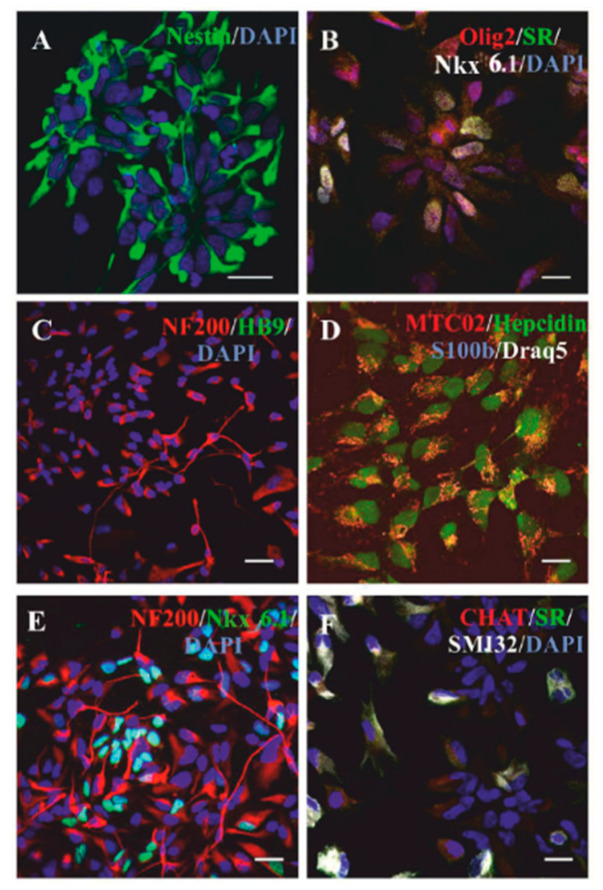
Neural progenitors derived from induced pluripotent cells (NP-iPS) in vitro characteristics. Immunostaining shows that NP-iPS cultures that were used for transplantation at the starting point of differentiation (contained nestin- (**A**), Olig2-, serotonin-(SR), and Nkx6.1-positive cells (**B**). At day 0, NP-iPS cultures were NF200-positive, but they did not express a motoneuron (MN)-specific marker HB9 (**C**). All NP-iPS were positively stained with human mitochondria (MTC02) and expressed a hepcidin (**D**), but they were S100β-negative (**D**). NF200 and Nkx6.1 co-staining revealed equal proportions of Nkx6.1-positive and Nkx6.1-negative cells in cultures (**E**). NP-IPS cultures did not contain acetylcholine transferase (CHAT)-positive cells, and only few cells were SMI32-positive (**F**). Scale bars = 20 µm.

Our group has previously characterized NP-iPS prior to and after pre-differentiation, and we showed that after seven days in culture, these cells do not express pluripotent markers (nanog, SSEA-4, and TRA-1-60), and the expression of OCT-3/4 is significantly decreased (less than 30%); however, more than 80% of the cells express neural surface markers: CD133, CD24, CD29, CD56, PSA-NCAM, NF70, as evaluated by FACS analyses. The pre-differentiation of NP-iPS led to a decrease in the expression of genes encoding neurotrophic factors VEGF and GDNF and an increase in the expression of BDNF and NT-3. Moreover, it also causes a shift in the cell population from glial to neuronal precursor, decreasing A2B5 expression according to FACS analysis, as well as decreasing *Gfap* and *S100b* gene expression and the increasing expression of neuronal genes (*Tubb3, Mapt, Syp, Hb9, Chat*) according to qPCR [17]. When transplanted into rats with spinal cord injury, we observed differentiation into more mature neuronal markers, such as serotonin-positive (SR) or acetylcholine transferase-positive (ChAT) cells [17]. In addition to the previously mentioned markers, here, we examine the cells for the presence of nkx6.1 (nkx6.1 is a crucial protein for the development and ventral neural patterning of MNs encoded by eponymous homebox gene [25]), SMI32, and NF200 (neurofilament markers). Hepcidin, a peptide hormone, plays an important role in systemic iron homeostasis [29]. It is a small protein (2.8 kDa), which is transported via platelets and exosomes, expressed in stem cells, involved in neural differentiation, and participates in glial scar formation [30,31]. In this study, we confirmed the neural phenotype of pre-differentiated NP-iPS precursors (day 0) prior to administration (Figure 1). We found that all cultured cells at day 0 were nestin-positive (Figure 1A) [32], but fewer cells expressed MN or MN precursors specific markers: NF200, SMI32, Olig 2, or Nkx6.1 (Figure 1B,C,E,F). Interestingly, we found that at the stage of neural progenitors, all cells expressed hepcidin, which has been also shown to be expressed in glial and oligodendrocyte precursor cells (Figure 1D) [33]. However, at day 0, we did not find any GFAP-positive cells in the culture, which also confirms our assumption about the shift to the neuronal population after pre-differentiation.

Cells were further differentiated toward neuronal phenotype (Figure S1), and more information on differentiation and their electrophysiological properties (Figures S2 and S3, Table S1) has been described in the Supplementary material.

### 2.2. NP-iPS Transplantation Improves Motor Activity and Extends the Survival of SOD1 Rats

**Figure 2:**
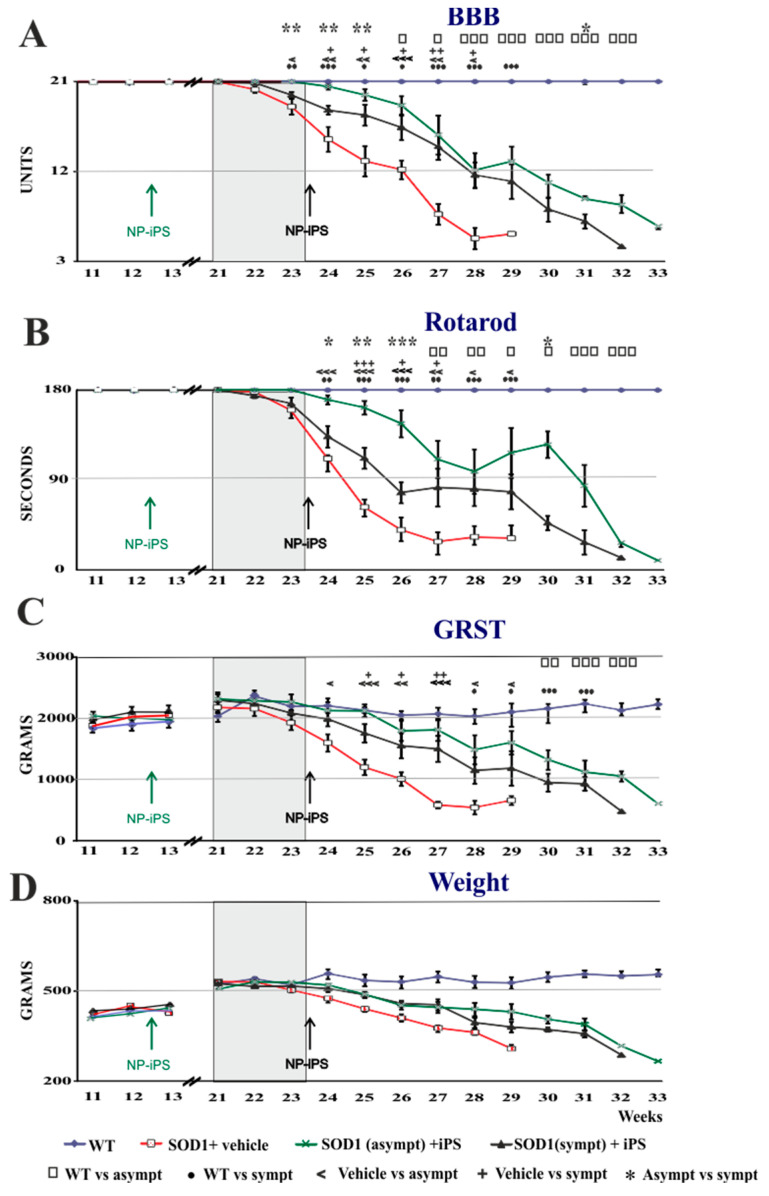
Intraspinal NP-iPS transplantation delays loss of motor function and prolongs the survival of SOD1^G93A^ transgenic rats. Human NP-iPS significantly delayed motor impairment evaluated by BBB- (**A**) and Rotarod-tests (**B**), and it slowed down loss of muscle strength assessed by the grip strength test (**C**) and demonstrated the tendency to protracted body weight loss compared with vehicle-treated littermates (**D**). Arrows depict time of cell injections into asymptomatic (green) and symptomatic (black) SOD1^G93A^ rats. Differences in the groups were analyzed by two-way ANOVA with repeated measures: statistical significance at *p*-values under 0.05 (*, □, ●, <, +), *p* < 0.01 (**, □□, ●●, <<, ++) and *p* < 0.001 (***, □□□, ●●●, <<<, +++). □ WT vs. asympt, ● WT vs. sympt, < vehicle vs. asympt, + vehicle vs. sympt, * asympt vs. sympt. A detailed statistical analysis of the behavioral changes between the groups is presented in Table A1 and Table A2.

**Figure 3:**
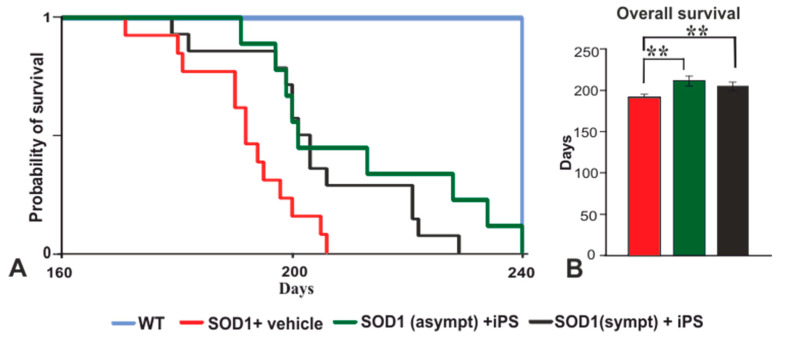
Animal survival. Kaplan–Meier plot describes the cumulative probability of animals’ survival in tested Tg and WT groups (**A**). NP-iPS cell therapy significantly extended the survival of both cell-transplanted groups (**B**). Differences in the groups were analyzed by one-way ANOVA, *p* < 0.01 (**).

We next assessed the ability of transplanted NP-iPS cells to influence behavioral decline and mortality in SOD1 rats. The stage of disease was established by Basso, Beattie, and Bresnahan (BBB), grip strength, and rotarod (Figure 2). We selected two groups of animals for intraspinal transplantation: asymptomatic (3 months old) or symptomatic (5.5–6 months old). The SOD1 rats were immunosuppressed and received multiple intraspinal (T10–11) transplants of NP-iPS as neural precursors. Control vehicle-treated SOD1 rats were injected with PBS at week 24. Each rat from the cell-treated groups (asymptomatic and symptomatic) received a total 3 × 10^5^ cells via six intraspinal injections (5 × 10^4^ cells per 1 µL), whereas vehicle-treated animals received six injections of PBS (1 µL/injection). The transplantation of asymptomatic rats with NP-iPS (group I) delayed the disease onset. While there were no differences at the time of first motor deficiency manifestation between symptomatic NP-iPS-treated (group II) or PBS-treated (group III) rats (Figure 2A–D), the transplantation of NP-iPS into asymptomatic animals (group II) significantly extended the time before the first manifestation of motor deficiency and significantly slowed disease progression when compared with iPS- or vehicle-treated SOD1 littermates (group II and III) (Figure 2A–D; Table A1). The asymptomatic NP-iPS-treated rats (group I) demonstrated a loss of motor activity (BBB), muscle strength (grip strength), and coordination (rotarod) that differed from WT control animals (group IV) at week 26 (Figure 2A), week 27 (Figure 2B), and week 30 (Figure 2C), respectively, while the vehicle SOD1 animals (group II) had significant deficits in all three tests by week 23 (Figure 2A–C, Table A1). The rate of progression of motor function loss in the symptomatic NP-iPS-treated group II compared with the vehicle-treated SOD1 group III was significantly slowed starting from week 24 (i.e., one week after the intraspinal transplantation of the NP-iPS) and further throughout the course of the disease (Figure 2A–D; Table A2). Differences in motor activity between the two NP-iPS-treated groups (I and II) of animals gradually started to diminish from the second week after transplantation to the symptomatic group II, and from week 27, both groups showed comparable motor deficit in functions. The average lifespan of the vehicle-treated SOD1 group III was 195 ± 2.8 days, while animals from group I survived for 214.4 ± 6.4 days and animals from group II survived for 208 ± 2.7 days. Thus, rats that were treated with human NP-iPS cells either at the asymptomatic or symptomatic stages (groups I and II) had significantly longer survival by 19 and 13 days, respectively, compared with vehicle-injected rats (Figure 3A,B). These results showed that intraspinal transplants of NP-iPS cells preserve motor function and prolong survival in SOD1 rats.

### 2.3. Spinally Grafted NP-iPS Are Neuroprotective and Do Not Cause Tumors

**Figure 4:**
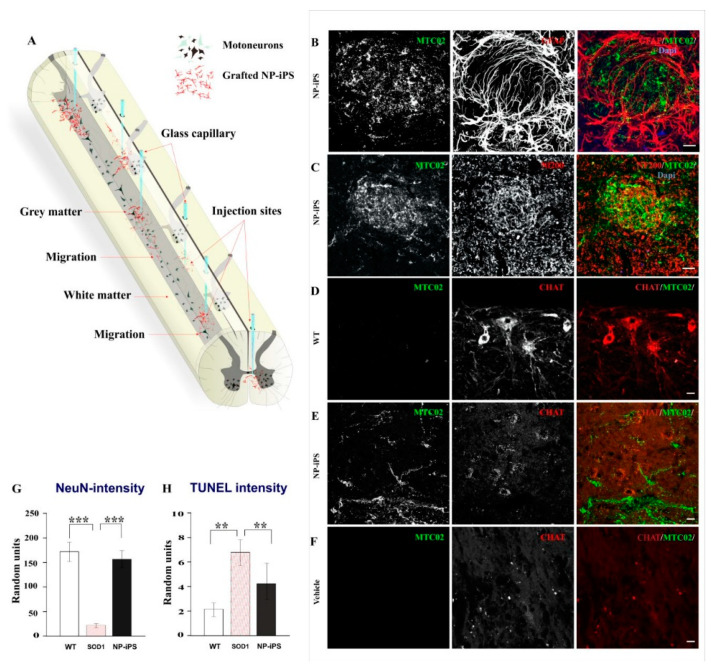
Survival and engraftment of NP-iPS. Schematic diagram shows a map of intraspinal injection sites (via the glass capillary tubes) and distribution of the grafted cells within the spinal cord (**A**). Intraspinally transplanted NP-iPS engrafted into the host tissue at the site of injection (**B**), Engrafted cells also preserved the expression of host neurofilament (**C**). Compared to WT rats (**D**), cell-treated animals (**E**) had weaker acetylcholine transferase (CHAT) staining, even though NP-iPS engrafted in the close vicinity to host MNs (**E**); however, NP-iPS-treated rats still maintained CHAT-positive cells (**E**) compared with vehicle-treated animals, where the CHAT staining was almost undetectable (**F**). A neuroprotective effect evaluated by NeuN fluorescence intensity revealed a significantly higher fluorescence in the ventral horns of NP-iPS-treated superoxide dismutase (SOD1)-rats compared with the vehicle-treated SOD1 littermates (**G**). TUNEL staining confirmed less apoptosis in NP-iPS-treated SOD1-rats compared with the vehicle-treated SOD1 littermates (**H**). Cell nuclei were visualized with 4′,6-diamidino-2-phenylindole (DAPI) staining. Differences in the groups were analyzed by one-way ANOVA, *p* < 0.01 (**), *p* < 0.001 (***). Scale bars = 20 µm.

**Figure 5:**
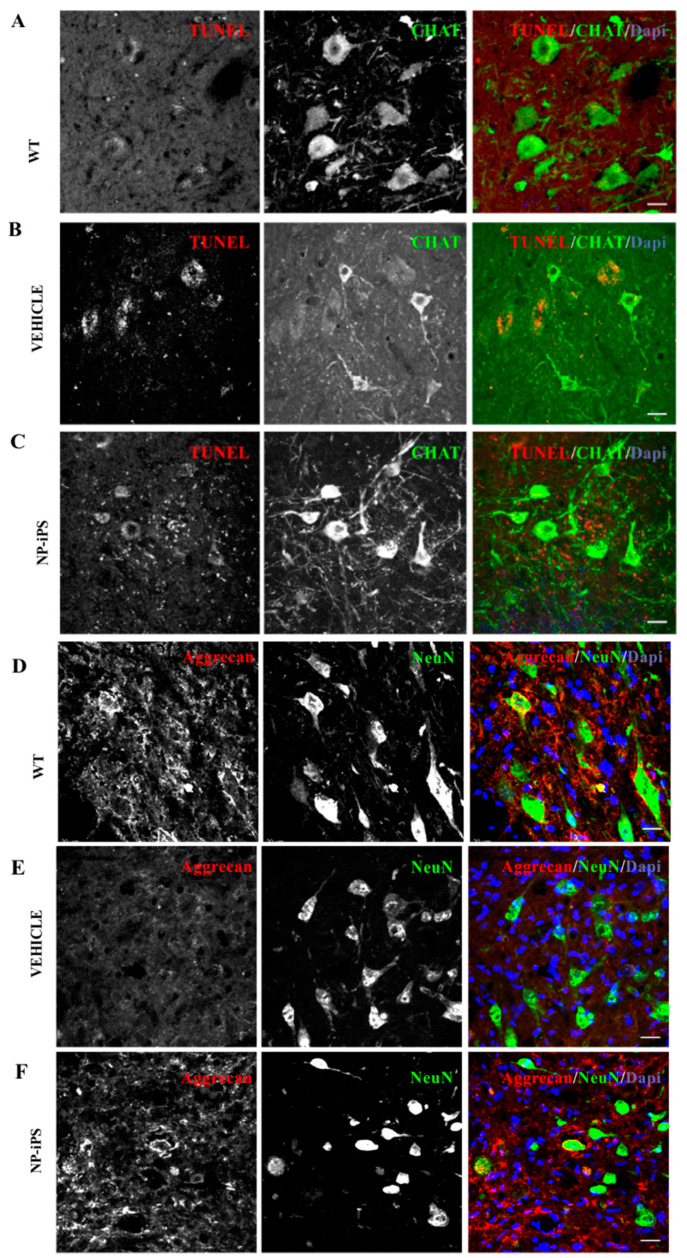
Transplantation of NP-iPS preserved spinal perineuronal nets. Co-localization of TUNEL and anti-CHAT staining confirmed MN apoptosis in vehicle-treated SOD1 rats (**B**), which was not seen in WT animals (**A**) and was faintly detected in NP-iPS treated animals (**C**). IHC staining demonstrates the normal perineuronal nets (PNN) structure revealed by anti-aggrecan antibody in the ventral horns of WT rats (**D**). NP-iPS-treated rats (**F**) had a similar expression of PNN to that observed in WT littermates (**D**), while vehicle-treated SOD1 Tg animals had mitigated PNN structure (**E**). IHC staining revealed the high power of preserved aggrecan protein in close vicinity to engrafted NP-iPS (**F**), while vehicle-treated Tg rats showed vestigial PNN (**E**). Cell nuclei were visualized with DAPI staining. Scale bars = 20 µm.

To study the survival, fate, and neuroprotective effect of grafted human NP-iPS, we immunostained spinal cord tissue using a combination of anti-human MTC02 antibody with antibodies of interest. MTC02 antibody stains specifically human mitochondria and is often used as a specific marker of human cells [17,25]. Animals were euthanized at week 34 (22 weeks after the injection of the asymptomatic group I and 10 weeks after injection of the symptomatic group II). The distribution of intraspinally grafted cells within the spinal cord (T10-11) and routes of delivery are shown in the schematic diagram (Figure 4A). Immunostaining revealed a robust survival of NP-iPS cells in the symptomatic rats from group II (Figure 4B,C,E), whereas the spinal cord of asymptomatic animals (group I) no longer contained MTC02-positive cells, despite immunosuppression for the entire experimental period. Transplanted cells were found at the sites of injections (Figure 4B), and they had also migrated along the spinal cord axis. Unlike in acute spinal cord injury, grafted cells did not express the astrocytic marker GFAP or the neuronal cytoskeletal markers (neurofilament subunit 200 kDa (Figure 4C), and none of the grafted cells expressed a motoneuron-specific marker (CHAT) (Figure 4E). We looked for hyper-proliferation activity, rosette formation, or delineated tumors of human origin (staining for MTC02) and we did not observe any of these structures in any of the transplanted animals. We found that cell-treated animals (group I and II) had significantly better preserved MNs. Interestingly, transplanted NP-iPS often engrafted in the close vicinity to host MNs (Figure 4E). Cell-treated animals (Figure 4E) had 12% fainter spinal CHAT staining compared with WT rats (Figure 4D), but they were 15% more preserved than the vehicle-treated SOD1 littermates (Figure 4F). A quantitative evaluation of fluorescence intensity staining from the ventral horns demonstrated significantly stronger staining with the neuronal marker NeuN, localized mostly to motoneurons in NP-iPS-treated groups of animals compared with the vehicle-treated SOD1-Tg rats (Figure 4G; *p* ≤ 0.001). Less apoptosis was detected by TUNEL (transferase (TdT)-mediated dUTP nick end labeling) assay in NP-iPS-treated animals compared with the vehicle-treated SOD1-Tg rats (Figure 4H). Better motoneuron survival was also confirmed by co-localized staining for CHAT and TUNEL; we detected much less apoptosis in CHAT+ cells in NP-iPS grafted animals (Figure 5C) when compared to vehicle-treated SOD rats (Figure 5B). Hardly any apoptosis was found in WT littermates (Figure 5A). The Immunohistochemical (IHC) staining of WT spinal cords with anti-aggrecan antibody typically revealed PNNs wrapping healthy MNs (Figure 5D). However, in the SOD1-transgenic rats, IHC aggrecan staining in the ventral horn was absent at the terminal stage of the disease (Figure 5E). NP-iPS transplantation preserved aggrecan+ PNN staining (Figure 5F). These data showed that transplanted NP-iPS cells enable motoneuron survival and preserve their specialized extracellular matrix PNNs in SOD1 animals.

### 2.4. NP-iPS Graft Modifies Spinal PNN-, Neurotrophic-, and Apoptosis-Related Gene Expression

**Figure 6:**
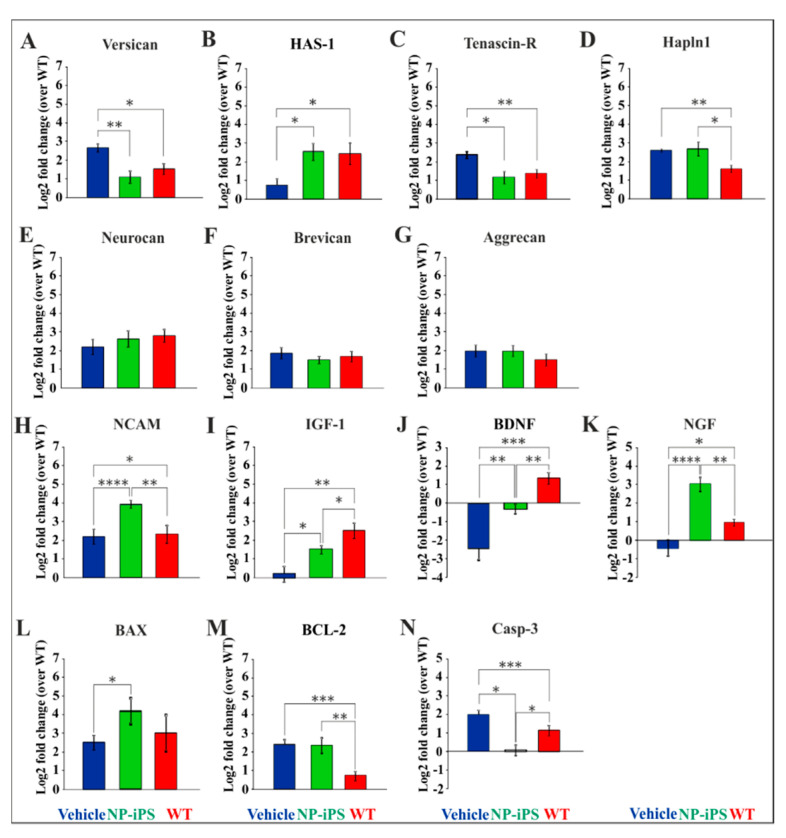
NP-iPS transplantation normalized the altered expression of spinal host genes. Seven PNN-related molecules of the ECM were tested. Vehicle-treated SOD1 rats had a significant dysregulation of *versican* (**A**), *has-1* (**B**), *tenascin-R* (**C**), and *hapln1* (**D**), which were recovered by NP-iPS transplantation. On the other hand, *neurocan* (**E**), *brevican* (**F**), and *aggrecan* (**G**) expression did not vary between the groups. Similarly, a significant dysregulation of genes encoding *ncam* (**H**) and growth factors *igf-1* (**I**), *bdnf* (**J**), and *ngf* (**K**) was amended by NP-iPS. Cell-treated rats had significantly upregulated *bax* compared to vehicle-treated littermates (**L**). Both vehicle and cell-treated animals have upregulated apoptosis-related gene *bcl-2* (**M**), while *caspase-3* (**N**) was downregulated by the delivery of NP-iPS (**N**). Bars represent the means ± SEM of the signal values assessed by the 2^−ΔΔCt^ method normalized to two endogenous reference genes, which were recalculated to relative quantities and transformed to a log2 scale. Differences in the groups were analyzed by one-way ANOVA: statistical significance at *p* values under 0.05 (*), *p* < 0.01 (**), *p* < 0.001 (***) and *p* < 0.0001 (****).

We studied the host-derived mRNA expression levels of PNN-, apoptosis-, and neurotrophin-related genes in the lumbar spinal cord of SOD1 and WT animals. *Versican, has-1, and tenascin-R* had similar expression in cell-treated SOD1 animals (group A) and WT animals (group C; Figure 6A–C), while vehicle-treated SOD1 rats (group B) had upregulated *versican* and *tenascin-R* (Figure 6A,C) and downregulated *has-1* (Figure 6B).

*Hapln1* was upregulated in both SOD1 groups (NP-iPS- and vehicle-treated) when compared to WT littermates (Figure 6D). No changes were found in expression of *neurocan, brevican*, and *aggrecan* (Figure 6E–G). The *Ncam* gene was significantly overexpressed only in NP-iPS injected animals (Figure 6H). Downregulation in the expression of *igf-1, bdnf*, and *ngf* genes was observed in SOD1 animals compared to WT littermates, which was partially rescued by the application of NP-iPS (Figure 6I,J). In contrast, a significant overexpression of the *ngf* gene was detected in cell-treated SOD1-rats when compared to both vehicle-treated SOD1 animals and WT (Figure 6K).

We also observed that an application of NP-iPS led to a significant upregulation of the *bax* gene and downregulation of *casp-3* gene in SOD1–Tg rats (Figure 6L,N). Both NP-iPS-treated and vehicle-treated rats had significantly upregulated levels of the *bcl-2* gene compared to WT animals (Figure 6M).

### 2.5. Intraspinally Grafted NP-iPS Normalizes Expression of PNN-Related Chondroitin Sulfate Proteoglycans in Terminal SOD1 Rats

**Figure 7:**
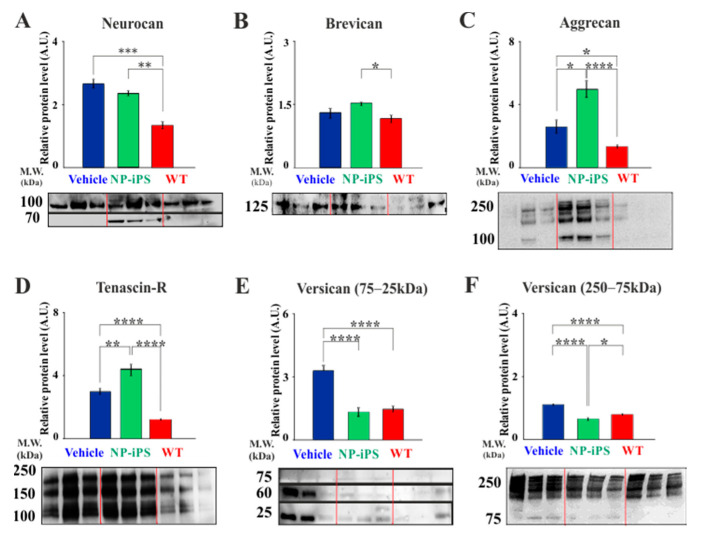
Identification of PNN-related molecules contributing to the disorganized spinal PNN structure. Immunoblotting of gel filtration fractions for the presence of the spinal PNN-related ECM molecules identified the upregulation of neurocan (**A**), aggrecan (**C**), tenascin-R (**D**), and both versican fractions 75–25 kDa (**E**) and 250–75 kDa (**F**); as well as a normal amount of brevican (**B**) in SOD1 animals compared with WT controls. NP-iPS-treated rats showed significantly higher amounts of ECM molecules (**A**–**D**) than in WT, except for versican (**E**,**F**), whose levels remained similar to those observed in WT littermates. Differences in the groups were analyzed by one-way ANOVA: statistical significance at *p* values under 0.05 (*), *p* < 0.01 (**), *p* < 0.001 (***) and *p* < 0.0001 (****).

**Figure A1:**
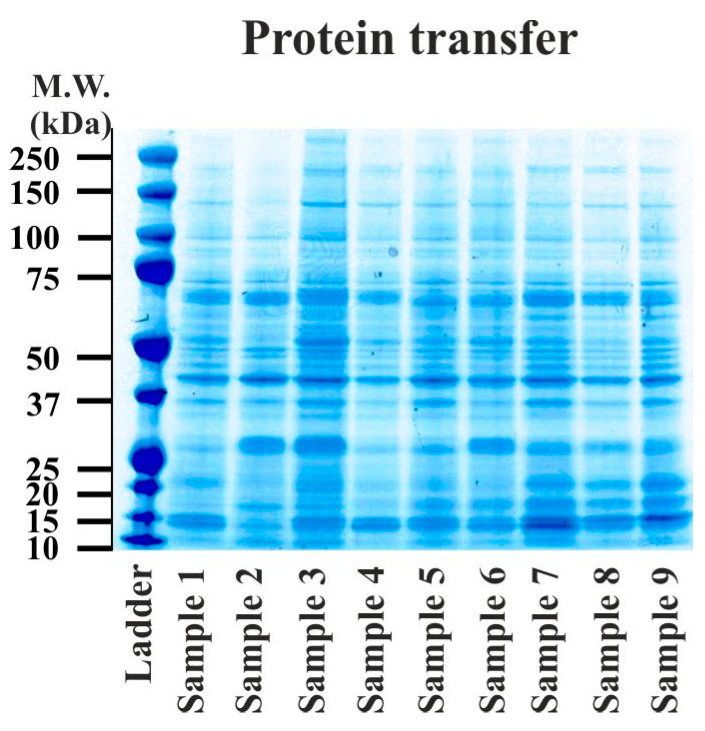
Coomassie staining of a PVDF membrane showed the homogeneity and completeness of the spinal gel filtration fractions transfer from the NuPAGE 4–12% Bis-Tris gel.

The ECM has a large influence on neuronal survival, and the specialized matrix structures PNNs have strongly protective properties [26,27]. We described above the preservation of PNNs around motoneurons by NP-iPS transplantation and further investigated whether neuroprotection by NP-iPS cells was associated with preservation of the ECM components of PNNs. We previously reported that mutant SOD1-transgenic rats at the terminal stage of disease had a disorganized structure of PNNs [21]. Therefore, we performed immunoblot analysis of PNN-related ECM molecules isolated from spinal cord homogenates from SOD1-transgenic rats at the terminal stage either treated with NP-iPS cells (group A) or vehicle (group B) (Figure 7). The immunoblot analysis revealed that the SOD1-transgenic vehicle- and NP-iPS-treated rats had a higher expression of neurocan and brevican compared to the WT age-related littermates (group C) (Figure 7A,B). A strong upregulation of aggrecan and tenascin R was observed in cell-treated animals when compared to vehicle-treated SOD1 and WT rats (Figure 7C,D). Considering that protein expression eluted broadly with apparent molecular weights between 250 and 25 kDa, we separately analyzed versican fractions with molecular weights between 250–75 kDa and 75–25 kDa. Quantitative analysis showed that vehicle-treated SOD1 rats had an upregulated expression of both versican fractions and that the application of NPs-iPS downregulated the expression of these versican molecules, so cell-treated animals had a similar expression of the aforementioned fractions as WT littermates (Figure 7E,F). After each immunoblotting, the quality of proteins transfer from gel to the membrane was controlled by Coomassie staining (Figure A1). These results revealed that protein levels of several ECM molecules that are found in PNNs are enhanced in SOD1 animals treated with NP-iPS transplants. In some cases, the protein levels are higher in transplanted animals than WT controls, suggesting that the transplants can elicit the production of ECM components.

## 3. Discussion

Progress in stem cell biology over the last decade has proven the great potential of the application of stem cell technologies, especially iPSC-based, in the search of the mechanisms and new therapies for incurable neurological diseases. The delivery of stem cells (SC) and progenitors has been shown to promote functional, behavioral, and morphological improvements, extend survival, and even structurally integrate into the segmental motor circuitry of mutant SOD1 animals [24,34,35,36,37,38]. The neuroprotective effects of SC after the transplantation have been related to the secretion of neurotrophic factors, immunomodulatory influence, microglia activation, stimulation of neurogenesis, and repopulation of the CNS with new neural cells, changing of the host gene and protein expression, the generation of microRNA, etc. [17,39,40]. In the present study, we confirm the neuroprotective effect of NP-iPS cells and study their effect on the expression of neurotrophic, inflammatory and ECM molecules. A particular focus has been the ability of the cells to preserve PNNs around MNs and influence the production of their constituent molecules. The implanted cells did not differentiate into MNs, but they remained undifferentiated. The environment of the SOD-1 spinal cord may lack the molecules that can drive precursors toward a more mature phenotype.

Previous work has demonstrated the protective and regenerative effects of transplanted NP-iPS cells. They have promoted spinal cord regeneration after experimental injury via the stimulation of neurotrophic factors expression; however, maturation and differentiation took four months [16,17]. Similarly, Popescu et al. also demonstrated that transplanted human iPSC-NPs can engraft, survive, and differentiate into mature neurons by 60 days after the cell delivery [23]. Other iPSC-derived neurons and astroglia generated from patients with familial ALS have been shown to recapitulate key aspects of disease pathology and biochemistry as well as ameliorate disease progression after transplantation [9,41,42]. The fate of the grafted cells is often different. Kondo et al. transplanted glial restricted progenitor cells derived from iPS cells into symptomatic ALS mice, and most of the grafted cells differentiated into mature astrocytes, while a relatively small population of grafts also differentiated into CNPase^+^ oligodendrocytes or MAP2^+^ neurons [9]. On the contrary, Popescu injected neural progenitors into asymptomatic ALS rats and reported motoneuron differentiation within 60 days [23]. In the present study, we revealed that NP-iPS cells transplanted either pre-symptomatically or after motor deficit signs have started have a robust effect on the preservation of motor behavior, survival of animals to their endpoint, and on the density of neurons in the ventral horn in the rat SOD-1 model. The grafted cells had a limited survival time after transplantation, and by the end of the experiment (2.5 months after administration in the symptomatic group and 5 months after administration of the pre-symptomatic group), surviving NP-iPS cells were only seen in the late-grafted animals. However, the preservation of behavior was seen in both groups. We speculate that the cell fate is most likely dependent on the neural progenitor phenotype. In addition, the environment of the SOD1-transgenic animals may negatively affect cell survival. We have observed worse survival of grafted human mesenchymal cells in SOD1 rats than in WT littermates [43]. Our data from earlier studies with NP-iPS in stroke or spinal cord injury confirmed robust survival and neuronal differentiation but not earlier than four months after transplantation [17,44]. Here, we observed that in the SOD1 environment, the graft–host interaction had a positive outcome in terms of lifespan and neuroprotection, but at the same time, we observed lower cell survival and no differentiation of the grafts toward neuronal phenotype. Since we did not inject WT animals with NP-iPS, it is difficult to interpret whether low cell survival is due to SOD1-mutated environment or not.

There are several mechanisms by which the cells mediate their protective effects. Kondo et al. demonstrated the differentiation of human iPSC-glia restricted NPs to neuronal phenotype, prolonged survival, and the improved motor activity of symptomatically treated animals accompanied by an increase in VEGF secretion [9]. A study published by Wyatt reported an upregulation of NGF, NT-3, and NT-4 but not VEGF [34]. Suzuki also reported on the important neuroprotective role of GDNF on MNs survival in a rodent model of ALS [45]. Lunn et al. demonstrated that increased IGF-I induces more potent MNs protection from excitotoxicity [46]. In the present study, we saw a significant loss of spinal *ncam*, *igf-1*, *bdnf*, and *ngf* mRNA expression in SOD1-transgenic rats, whereas the transplantation of NP-iPS partially normalized *igf-1* and *bdnf* expression compared with WT littermates, and *ngf* was expressed at a very high level. Considering that the aforementioned genes encode neurotrophins that are implicated in prenatal and postnatal events in CNS development (especially IGF-1) such as the control of gliogenesis, neurogenesis, neuron survival, differentiation, and may mediate PNN formation and plasticity, we could speculate that similar mechanisms are activated by the intraspinal transplantation of human NP-iPS [47]. We also measured the mRNAs related to cell death. We previously reported that DNA damage originated from the mitochondrial DNA and subsequently spreads to the nucleus, and that stem cell transplants protect against both [22,35,36]. Kostic et al. clearly showed that upregulated *bcl-2* and corresponding protein delays the terminal stage of disease in SOD1^G93A^ mice but does not affect the progression of the disease [48]. In the present study, we report that SOD1 animals have upregulated *bcl-2* and *caspase-3* and that intraspinal transplants of NP-iPS downregulated *caspase-3* but not *bcl-2.* We can speculate that the observed protective effect in cell-treated animals is related to the downregulation of caspase-3, which is responsible for the induction and execution of apoptosis [49]. In addition to neuronal cell death, the upregulation of *caspase-3* in SOD1 animals can also be related to astrocytes, since caspase-3 activation results in the truncation of EAAT2/GLT1 glia cell transporter, which is involved in ALS pathology [50]. Therefore, the cell-based therapy with *caspase-3* downregulation might positively influence astrocytes rather than just reduce apoptosis, since the *bax* gene was not downregulated. Further experiments and evaluation on protein level would support this speculation.

A novel aspect of our study has been a focus on the ECM and PNNs. A healthy ECM is an essential component of normal CNS functioning, and it affects neuronal survival. ECM deficits, the dysregulation of chondroitin sulphate proteoglycans (CSPGs), or aberrant PNN formation have been associated with several neurological diseases such as schizophrenia, autism, Prion’s disease, multiple sclerosis, human immunodeficiency virus dementia, stroke, spinal cord injury, and amyloidopathies [51,52]. While there are also reports showing that PNNs can be neuroprotective [26,27], only a few reports have suggested ECM involvement in ALS. Mizuno et al. demonstrated that the early symptomatic stage of ALS is accompanied by the upregulation of versican and phosphacan, and it is co-localized with reactive astrocytes especially around residual large ventral horn neurons [53]. An increased degradation of PNNs enwrapping motoneurons has been demonstrated by an administration of recombinant ADAMTS-4 accompanied by clinical signs of neuromuscular dysfunctions, the decreased expression of several neurotrophic factors in astrocytes and microglia, as well as increased vulnerability to the degeneration of lumbar MNs, which is most likely due to the loss of their ECM envelopes [54]. We reported earlier that PNNs are affected in the SOD1-transgenic rats and that the intrathecal application of hMSC into SOD1 rats had a significantly upregulated expression of the *hapln1*, *neurocan*, and *tenascin-R* genes. Quantification of IHC staining of aggrecan, versican, hapln1, and phosphacan did not reveal any differences between SOD1 controls and cell-treated animals [21]. In the present study, a detailed analysis of spinal CSPGs using SOD1-Tg rats confirmed a similar upregulation of *tenascin-R*, *hapln1*, *versican*, and *neurocan* genes, which was accompanied by a higher expression of tenascin-R, brevican, aggrecan, and neurocan proteins compared with WT age-related littermates. Interestingly, unlike hMSCs, transplanted NP-iPS normalized the expression of *tenascin-R*, *versican*, and *has1 genes*, and it caused the hyper-expression of aggrecan and tenascin-R. Since similar results were observed on the level of gene as well as protein expression, we can speculate that the breakdown of PNNs during disease progression may lead to an overexpression of ECM compounds as a result of defense reaction of the tissue. These results parallel ECM transformation during the critical period of CNS development when changes in core proteins and glycosaminoglycan sulfation patterns trigger PNN formation [28,55,56,57]. However, further experiments will be necessary to prove this hypothesis.

Transplanted cells can induce changes in cytokines and growth factors in the injected areas [58], which ultimately affect the metabolism of ECM molecules. We have observed the normalization of *igf-1* and *bdnf* and upregulation of *ngf* in cell-treated animals in this study and similar expression patterns in our previous study with NP-iPS in spinal cord injury [17]. Some of these factors are known to induce the expression of ECM molecules, e.g., TGF-beta, TNF-alpha, or Bdnf in CSPG synthesis, metalloproteinases in ECM degradations, and removal [59], and therefore, it releases the ECM-bound molecules to be recognized by neurons. In addition, otx-2 bound to PNNs will be released upon ECM degradation. As otx-2 is a soluble transcription factor, this may further alter gene expression in the surrounding neurons [60]. Due to the lack of NP-iPS grafted into WT animals, further studies are required to fully separate the influence of SOD1 environment on the changes in gene and protein expression from cell treatment.

## 4. Materials and Methods

### 4.1. Culture of Human Induced Pluripotent Stem Cell-Derived Neural Precursors

The early neural precursors used in our study were derived from the IMR90 cell-line generated by the transduction of female human fetal lung fibroblasts (ATCC, Manassas, VA, USA) with a cocktail of lentivirus-mediated human cDNAs (OCT4, SOX2, NANOG and LIN28). NP-iPS were induced in the growth media containing 500 ng/mL of Noggin (R&D Systems, Minneapolis, MN, USA); 10 nM transforming growth factor-β pathway inhibitor SB 431542 (Sigma-Aldrich, St. Louis, MO, USA); 10 μg/mL bFGF and 20 μg/mL BDNF (both from Pepro Tech, London, UK) [17,25,61]. A detailed description of the protocol, composition of growth media and solutions used for culturing the neural precursors were reported earlier by our group [25]. Briefly, NP-iPS were cultured in a medium composed of DMEM/F12 and Neurobasal media mixture (1:1) supplemented by B27 and N2 supplements (GIBCO, Life Technologies, Grand Island, NY, USA), l-glutamine (2 mM, Sigma-Aldrich), penicillin and streptomycin (50 U/mL, GIBCO), FGF (10 ng/mL), EGF (10 ng/mL), and BDNF (20 ng/mL) (PeproTech, London, UK). The media were replaced every second day. Prior to transplantation, NP-iPS were cultured for 7 days in the same medium, except for the omission of FGF and EGF factors.

### 4.2. Animals

Transgenic adult male Sprague–Dawley rats (SD-Tg SOD1^G93A^L26H) that overexpress human SOD1 and carry the Gly-93-Ala mutation (Taconic, Rensselaer, NY, USA) were used as an animal model of familial amyotrophic lateral sclerosis (fALS). The study involved a total of 88 rats, SOD1 and wild type (WT). The rats were bred in an animal facility and housed under standard laboratory conditions: a 12:12 h dark/light cycle, at 23 °C, two rats per cage, with food and water supplied ad libitum. Transgenic rats were bred and maintained as hemizygotes by mating transgenic male with WT female (Sprague–Dawley). Size of the litter varied between 12 and 16 pups, and usually, one-third were SOD1 positive. We used SOD1-positive animals as well as their wild-type (WT) littermates. To distinguish between WT and SOD1-positive rats, all animals were genotyped. Exogenous SOD1 transgene was detected in amplified pup tail DNA (extracted from 20-day-old animals) by polymerase chain reaction. This study was performed in accordance with the European Communities Council Directive of 22 September 2010 (2010/63/EU) regarding the use of animals in research, and approved by the Ethics Committee of the Institute of Experimental Medicine, Czech Academy of Science, Prague, project number 12/2013, 17/2013 and 127/2016.

### 4.3. Behavioral Testing

To evaluate the early stage, progression, and end stage of the disease, once a week (unless more frequent testing was required), an examiner scored the motor functions and body weight of the animals on the same day and time. Animals were trained to undergo a combination of motor tests (grip strength, BBB, rotarod), and “thirty seconds” test at the age of 3 months, and they were tested one month prior to the expected disease onset, and subsequently once a week throughout the course of the experiment. A short description of the used tests follows:

#### 4.3.1. Grip Strength Test (GrST)

The grip strength (GrST) test was used to assess the muscle strength. Animals were allowed to grasp a metal grid and then pulled backwards, on the horizontal plane, by the examiner. The maximum force generated by the rat just before losing its grip was recorded by a grip-strength meter (Grip Strength Meter BSGT2S, Harvard Apparatus, Holliston, MA, USA).

#### 4.3.2. BBB Test

The BBB test evaluates the motor activity by a unified scale originally developed by Basso to grade spinal cord injury and adapted by Forostyak et al. to evaluate ALS progression [22,62]. Trunk stability, forelimb–hindlimb coordination during gait, limb advancement, and paw placement were rated by a 21-point scale, with 21 indicating a full range of movement (healthy rat) and 0 indicating the total absence of limb movements.

#### 4.3.3. Rotarod

To test the animals’ balance, coordination, physical condition, and motor planning, animals were placed onto a rotating cylinder (RotaRod, Ugo Basile, Gemonio, Italy) that was mechanically driven at a constant speed of 15 rpm. Animals were allowed to run on the rotating rod for 180 s (five times with 5 min intervals). Once an animal was unable to complete the test twice in a row (typically between 160 and 170 s), it was regarded as disease onset. Then, animals were tested subsequently to analyze the disease progression, therapy effects, and diagnose the end stage of the disease.

#### 4.3.4. Thirty Second Test

The “thirty second test” was performed in addition to other behavioral tests and body weight measurements to confirm the end stage of ALS. An animal was placed on its side, and the time spent to position itself back into the original upright posture was measured. Failure in standing up within 30 s was considered as the end stage of the disease.

The following criteria were used to diagnose the disease onset: (i) BBB score drop from 21 to 17–16, (ii) grip strength decrease by more than 200 g compared to the individual baseline, (iii) rotarod drop from 180 to 160 s, or (iv) the rat started to lose body weight. Rats experiencing the first symptoms of motor deficiency (between 160 and 170 days of age) were observed for another five to seven days to confirm onset. The behavioral study involved a total of 43 rats. Animals were randomly divided into groups:

(I) NP-iPS transplantation into asymptomatic SOD1 rats (*n* = 9)
(II) NP-iPS transplantation into symptomatic SOD1 rats (*n* = 14)
(III) vehicle injection into SOD1symptomatic rats (*n* = 13)
(IV) vehicle injection into wild type (WT) littermates (*n* = 7)

The terminal stage of the disease was confirmed when rats expressed a drop of the following behavioral scores: decline in motor activity by 75% (BBB score drop to 5), decline in grip strength by 75% (grip strength decrease to 500 g), decline in rotarod by 95% (corresponded to 5–10 s), decline in body weight by 30% (up to 260 g), and additionally, they were unable to right themselves when placed on their side within 30 s. For ethical reasons, animals were ethically sacrificed after reaching the terminal stage.

### 4.4. Intraspinal Transplantation of NP-iPS

Starting from one day prior to intraspinal cells/vehicle administration, animals received immunosuppression daily with Sandimmun (10 mg/kg; Novartis Pharama AG, Basel, Switzerland), Immuran (4 mg/kg; GlaxoSmithKline, Brentford, UK) and Solu-Medrol (2 mg/kg; Pfizer, Puurs, Belgium). NPs-iPS were grafted intraspinally at pre-symptomatic (3 months old) and early symptomatic (5.5–6 months old) stages of the disease. NP-iPS were cultured for 7 days after thawing until they reached 80–90% confluency. When the desired confluence was reached, NPs-iPS were washed with media, re-suspended, and counted (in Bürker counting chamber per 1 μL of cells suspension). Cells were also assessed for cell viability (not lower than 90%) by a trypan blue method before transplantation. The cells were suspended at a concentration of 5 × 10^4^ cells/μL in PBS and were intraspinally (T10-11) injected by the protocol described earlier [22]. Since this protocol has already shown improvement in previous behavioral study [22], we have chosen the same strategy in this study. Briefly, the rats were anesthetized with isofluorane (3%) vapor inhalation in air (Forane, Abbot Laboratories, Ltd., Queenborough, Great Britain). The spinal cords of animals from group I + II were exposed at the T10-11 levels, followed by six intraspinal injections in a chessboard manner: three injections on the left and three on the right side (diagonally), with a distance of three millimeters between the injection sites (Figure 4A). Each injection contained 5 × 10^4^ NP-iPS; thus, every rat received a total of 3 × 10^5^ cells. The cells were injected into the ventral horns of the spinal cord by means of a stereotactic apparatus with the following coordinates: a depth of 3 mm from the dorsal surface and 2 mm laterally from the midline on the left and on the right. The injections were made via an attached glass capillary tube at a rate of 1 μL/min using a Nano-Injector (Stoelting Co., Wood Dale, IL, USA). To prevent the reflux upon glass capillary tube withdrawal, the glass capillary tube was kept in place for an extra minute. After the wound suturing, all animals were returned to their home cages, where they received food and water ad libitum. The control group of animals (III and IV) underwent exactly the same procedures as those from the cell-treated groups, only by replacing the cell suspension by the same volume of vehicle (PBS). Surgeries were performed under aseptic conditions by means of an OPMI-1 surgical microscope (Carl Zeiss, Oberkochen, Germany).

### 4.5. Postoperative Care

After surgery, all animals received an intramuscular (0.3 mL) bolus injection of Ampicillin (Biotika a.s., Slovenská Ľupča, Slovakia) and a subcutaneous (0.1 mL) injection of Rimadyl (Pfizer, Puurs, Belgium). All rats were immunosuppressed by the aforementioned combination of drugs: Sandimmun, Immuran, and Solu-Medrol lifelong.

### 4.6. Tissue Processing and Immunohistochemistry

Animals from groups I–IV were over-anesthetized with an intraperitoneal injection of ketamine (Narketan 10%, 50 mg/kg)/xylazine (Rometar 2%, 6 mg/kg) and transcardially perfused with 0.1 M saline in combination with a 4% paraformaldehyde (PFA). Spinal cords were dissected from the vertebral canal. The spinal cord segments grafted with either the NPs-iPS or vehicle, together with the nearby tissue (Th7-Th13), were post-fixed in 4% paraformaldehyde solution, cryo-protected in a gradient of sucrose solutions (10–30% in 0.2 M phosphate buffer) and longitudinally sectioned (group I, *n* = 9; group II, *n* = 14; group III, *n* = 13; group IV, *n* = 7). Longitudinal sections were used to study the fate of the transplanted cells (IHC staining for human-specific mitochondrial marker, MTC02) and comparative analysis with vehicle-treated tissues. Sections were blocked with a 3% goat serum and 3% bovine serum albumin in Tris buffer with 0.2% Triton-X100 (Sigma-Aldrich), which was followed by incubation with primary and appropriate secondary antibodies (Table A3). PNNs were visualized by biotinylated *Wisteria floribunda* agglutinin (WFA, 20 μg/mL, Sigma-Aldrich) labeled with streptavidin conjugated to Alexa488 (Molecular probes, Eugene, OR, USA, 1:100). To visualize the cell nuclei, sections were either stained with 300 nM 4′,6-diamidino-2-phenylindole (DAPI) or 1 µM 1,5-bis{[2-(di-methylamino)ethyl]amino}4,8-dihydroxyanthracene-9,10-dione (Draq5), after washing with PBS, and mounted with Aqua-Poly/Mount (Polysciences, Warrington, PA, USA). The sections were evaluated using a Zeiss LSM 5 DUO (Zeiss, Oberkochen, Germany) confocal microscope equipped with an Ar/HeNe laser.

The spinal cord segments located cranially and caudally (T5-7 and L1-L3) from the transplantation area were dissected and placed either in a RNAlater Stabilization Solution (Thermo Fisher, Waltham, MA, USA) or in a solution containing a cocktail of protease (cOmplete^TM^ ULTRA tablets, mini, EASYpack, Roche, Mannheim, Germany) and phosphatase inhibitors (Thermo-Scientific Pierce Phosphatase Inhibitors mini tablets), homogenized, and stored for further rt-qPCR (group A (SOD1 + NP-IPs), *n* = 8; group B (SOD1 + vehicle), *n* = 8; group C (WT), *n* = 8) or WB analysis (group A (SOD1 + NP-IPs), *n* = 5; group B (SOD1 + vehicle), *n* = 8; group C (WT), *n* = 8), respectively. Since the functional outcome at the end stage of the disease was the same in asymptomatic and symptomatic cell-treated rats, we pooled both groups to obtain a sufficient sample size.

### 4.7. Quantification of Motoneuron Staining in the Spinal Ventral Horns

To evaluate a neuroprotective effect of NP-iPS-based therapy at the end stage of the disease, the intensity of NeuN-positive staining was quantified from the ventral horns at both the thoracic and lumbar spinal cord levels. We have used this method of evaluation rather than an unbiased stereological method on serial sections, since the spinal cords were cut longitudinally, and we were unable to perform accurate calculations of MN numbers in all groups of animals. Briefly, ventral horn neurons were visualized using rabbit anti-NeuN primary antibody and Alexa Fluor 488 conjugated anti-rabbit IgG secondary antibody. Four images from spinal regions of interest (ROI, 100 µm^2^ each) were randomly imaged from the ventral horns area (two lefts and two rights) at the same level to the injection site (NPs-iPS or vehicle). The optical densities (gray-scale levels of the corresponding pixels of the pre-processed image) along with the surface area were determined by means of ImageJ software. The background optical density, calculated from a spinal cord sections processed without the addition of a primary antibody, was measured and subtracted. Then, all fluorescence intensity values obtained were normalized to the lowest intensity density in the vehicle-treated group (recorded intensity density of the ROI divided by the lowest intensity density of vehicle-treated ROI). We statistically evaluated four rats (5 slices each) that were randomly selected from each group. Images were taken by means of a Zeiss Axio Observer microscope with a 20× objective and Axio Vision4 software (Carl Zeiss Vision GmbH, Germany). The mean of the group was obtained from 16 ROIs (4 ROI per animal).

### 4.8. Immunocytochemistry

Cells plated onto laminin- and poly-l-ornithine-coated coverslips were fixed and immunostained according to the protocol described previously [16]. The primary antibodies used in the study are listed in Table A3. NPs-iPS blocked with normal goat serum and incubated only with secondary antibodies served as a negative control. Cell nuclei were stained by incubation with DAPI in PBS (5 min) at 24 °C, mounted with Aqua Poly/Mount and imaged using a ZEISS LSM 510 DUO confocal microscope (Zeiss, Oberkochen, Germany).

### 4.9. Electrophoresis and Western Blotting (WB)

Homogenates from freshly isolated spinal cord tissues were prepared by incubating the small spinal cord pieces in cOmplete^TM^ Lysis-M buffer (cOmplete^TM^ Lysis-M EDTA-free, Roche) containing cOmplete^TM^ protease and phosphatase inhibitors and following the manufacturer’s protocol. The homogenates were incubated on ice for 10 min before being centrifuged at 14,000× *g* (10 min, 4 °C), and the supernatant was extracted. Protein concentrations were measured by BCA Protein Assay Kit (Pierce, Rockford, IL, USA). We used 8 mg of proteins from each homogenate for the electrophoresis. Prior to electrophoresis, samples underwent chABC digestion using 25 lU/mL of enzyme and incubated for 3 h at 37 °C. Protein electrophoresis was carried out in a 4–12% Nu-PAGE Bis-Tris gels under reducing conditions with NuPage LDS sample buffer and Sample Reducing agent (NOVEX Invitrogen by Lifetechnologies^TM^, Carlsbad, CA, USA). Proteins were transferred to Invitrolon^TM^ PVDF membrane (Invitrogen by Lifetechnologies^TM^, USA) at 4 °C for 3 h at 100 V. After the transfer of proteins to the membrane, the quality of electrophoretic separation and uniformity of proteins transfer was controlled by the Ponceau S solution (Sigma, St. Luis, MO, USA). After visualization, the protein bands were destained by washing the membranes (twice) in TBS containing 0.1% Tween-20 (TBS/T) solution. Subsequently, the membranes were blocked with 5% skimmed milk (Marvel, Peterborough, UK) in TBS/T. Then, the blot was incubated overnight at 4 °C in TBS/T (with 1% *w*/*v* dried milk powder) containing antibodies against the protein of interest. A complete list of antibodies used in this study is given in Table A3. Membranes were washed three times in TBS/T and incubated for 2 h in TBS/T containing peroxidase-conjugated anti-mouse, anti-rabbit or anti-goat antibodies, depending on the species of primary antibody (1:10,000, Vector). Membranes were washed three times, and proteins of interest were visualized using a chemiluminescent substrate (Pierce™ ECL Western Blotting Substrate, ThermoFisher Scientific, Waltham, Ma, USA) and Alliance instrument (UVITEC Cambridge, Cambridge, UK). Data were analyzed and quantified using ImageJ.

### 4.10. Gene Expression

Experiments studying the gene expression levels were performed as described earlier [21]. The total amount of mRNA was extracted from the terminal spinal cords isolated from SOD1 rats (vehicle- and NP-iPS-treated) and healthy WT littermates of the same age using the High Pure RNA Paraffin Kit (Cat. No. 03270289001; Roche, Germany). The concentration of the total amount of mRNA was measured using a Nano Drop Spectrophotometer (Nano Drop Technologies, Wilmington, DE, USA). RT-qPCR was performed with reagents and kits purchased from Applied Biosystems (Foster City, CA, USA). First-strand cDNA was synthesized in duplicate from 10 ng of mRNA (20 µL reaction volume) by means of a High Capacity cDNA Reverse Transcription Kit (Cat. No.4368813) and pre-amplified using a TaqMan PreAmp Master Mix (Cat. No.4384267; Applied Biosystems, Waltham, MA, USA). Pre-amplification products were diluted 20 times in Tris-EDTA (TE) buffer as recommended by the manufacturer and subsequently used to quantitatively compare the gene expression of the PNNs components (*has-1*, *brevican*, *versican*, *aggrecan*, *tenascin-r*, *neurocan*, *hapln1*), growth factors (*ngf*, *igf-1*, *bdnf* and *ncam*), and apoptosis-related genes (*caspase-3*, *caspase-7*, *bcl-2* and *bax*). The RT-qPCR was carried out in a final volume of 10 μL. The amount of the PCR product was determined using the StepOnePlus^TM^ Real Time PCR System (Applied Biosystems), TaqMan Gene Expression Master Mix (Cat. No. 4369510), and TaqMan Gene Expression Assays (Cat. No.4331182) for *Rattus norvegicus* (Table A4). Thermal profiles were set as recommended by the manufacturer. All results were obtained using the integrated StepOne^TM^ Software (version 2.3, TermoFisher Scientific,) and further assessed through the 2^−ΔΔCq^ method normalized to two appropriate endogenous reference genes selected by NormFinder: *gapdh/Rn01775763_g1/* and *beta-Actin*/Rn00667869_m1. Gene expression was normalized against healthy WT littermates of the same age and compared between the groups. Finally, all data were recalculated to relative quantities and transformed to a log2 scale according to the MIQE (Minimum Information for Publication of Quantitative Real-Time PCR Experiments) recommendations [63].

### 4.11. Statistical Analysis

All numeric data are presented as mean values and analyzed with one-way ANOVA except for the behavioral results where two-way ANOVA with repeated measures was used (Sigma-Plot 9.0, Systat Software, San Jose, CA, USA). Group differences are presented as mean ± standard error of the mean (s.e.m.), while RT-qPCR results were calculated as described earlier for the 2^−ΔΔCt^ method and the Kaplan–Meier method was used to determine the difference in survival possibility rate between the groups of NP-iPS-treated (asymptomatic and early symptomatic) and vehicle-treated animals. Statistical significance was established at *p* values under 0.05 (*), *p* < 0.01 was considered as of a high statistical significance (**), *p* < 0.001 was considered a very high statistical significance (***) and *p* < 0.0001 was considered an extremely high statistical significance (****).

## 5. Conclusions

Taken together, our results show that NP-iPS cells transplanted into SOD-1 rats can surround motoneurons, protect them from degeneration, and preserve motor function. Their effects include the upregulation of neurotrophins and preservation of the ECM and particularly the PNNs. Our findings confirm that stem cell and progenitor therapy can modify the progression of neurodegenerative pathology. This might be combined with drugs that stop the loss of PNN or block the interaction of MIF with its known receptor (CD74) for a therapeutic strategy to treat ALS and other neurodegenerative diseases [64,65,66]. However, our results did not confirm the differentiation into any desired mature neuronal phenotype, and thus, the effect of NP-iPS is most likely paracrine and does not differ from the effects of non-neural stem cell applications such as MSCs.

## Supplementary Materials

Supplementary Materials can be found at https://www.mdpi.com/1422-0067/21/24/9593/s1.

## Abbreviations

acan	Aggrecan
ALS	Amyotrophic lateral sclerosis
BDNF	Brain derived neurotrophic factor
Bcan	Brevican
Casp-3	Caspase-3
Casp-7	Caspase-7
CSPGs	Chondroitin sulfate proteoglycans
EAAT2	Excitatory amino acid transporter2
EGF	Epidermal growth factor
ECM	Extracellular matrix
FGF	Fibroblast growth factor
GFAP	Glial fibrillary acidic protein
GRNP	Glial-rich neural precursor
GrST	Grip strength test
Has 1	Hyaluronan synthase-1
ICC	Immunocytochemistry
IHC	Immunohistochemistry
iPSC	Induced pluripotent stem cells
IGF-1	Insulin growth factor-1
hapln-1	Hyaluronan and proteoglycan link protein-1
MMP	Matrix metalloproteinase
ADAMST-4	Metalloproteinase with thrombospondin motifs four
MNs	Motoneurons
NF	Neurofilaments
NGF	Nerve growth factor
NP-iPS	Neural precursors derived from induced pluripotent stem cells
PNN	Perineuronal nets
SR	Serotonin
SC	Stem cells
SOD1	Superoxide dismutase-1
TnR	Tenascin-R
Tg	Transgenic animals
VEGF	Vascular endothelial growth factor
vcan	Versican
WB	Western Blot
WT	Wild type animal

## Appendix A

**Table A1:** Statistical analysis of behavioral tests between asymptomatically NP-iPS-treated group of animals and vehicle-treated littermates.

Weeks	BBB	Rotarod	GRST	Weight
23	***p* ≤ 0.0221**	*p* = 0.054	*p* = 0.08	*p* = 0.2
24	***p* ≤ 0.003**	*p* ≤ 0.0018	***p* ≤ 0.024**	*p* = 0.055
25	***p* ≤ 0.003**	***p* ≤ 0.0000004**	***p* ≤ 0.000061**	*p* = 0.10
26	***p* ≤ 0.003**	***p* ≤ 0.0000004**	***p* ≤ 0.000061**	*p* = 0.20
27	***p* ≤ 0.004**	***p* ≤ 0.0035**	***p* ≤ 0.000013**	*p* = 0.09
28	***p* ≤ 0.024**	***p* ≤ 0.04**	***p* ≤ 0.022**	*p* = 0.1
29	***p* ≤ 0.05**	***p* ≤ 0.014**	***p* ≤ 0.04**	*p* = 0.2

At week 23 (highlighted by gray color) SOD1-rats started to manifest a motor function deficiency, which was considered as a disease onset; animals in this group were treated with NP-iPS at week 12. Statistical significance between the groups are marked by bold font.

**Table A2:** Statistical analysis of behavioral tests between symptomatically NP-iPS-treated group of animals and vehicle-treated littermates.

Weeks	BBB	Rotarod	GRST	Weight
23	*p* = 0.2	*p* = 0.53	*p* = 0.37	*p* = 0.6
24	***p* ≤ 0.037**	*p* ≤ 0.21	***p* ≤ 0.053**	*p* = 0.2
25	***p* ≤ 0.021**	***p* ≤ 0.002**	***p* ≤ 0.011**	*p* = 0.5
26	***p* ≤ 0.02**	***p* ≤ 0.032**	***p* ≤ 0.04**	*p* = 0.2
27	***p* ≤ 0.0019**	***p* = 0.053**	***p* ≤ 0.0028**	*p* = 0.1
28	***p* ≤ 0.012**	***p* ≤ 0.011**	***p* ≤ 0.012**	*p* = 0.5
29	***p* ≤ 0.01**	***p* ≤ 0.026**	***p* ≤ 0.03**	*p* = 0.2

At week 23 (highlighted by gray color), SOD1-rats started to manifest a motor function deficiency; this was considered as a disease onset and animals were treated either with PBS or NP-iPS. Statistical significance between the groups are marked by bold font.

## Appendix C

**Table A3:** List of primary antibodies.

AB Name	Protein of Interest/Application	Species & Antibody Type	Concentrations	Manufacturer
AB1031	Aggrecan/WB, IHC	Rabbit Polyclonal	1:1000	Millipore, Burlington, MA, USA
12C5	Versican/WB, IHC	Mouse monoclonal	1:100	DSHB, Iowa city, IA, USA
Anti-Tenascin-R	Tenascin-R/WB, IHC	Goat polyclonal	1:1000	R&D Systems, Minneapolis, MN, USA
1F6	Neurocan/WB, IHC	Mouse monoclonal	1:100	DHSB
Anti-Brevican	Brevican/WB, IHC	Mouse polyclonal	1:100	Abcam, Cambridge, MA, USA
3F8	Phosphacan/WB, IHC	Mouse monoclonal	1:1000	DHSB
Anti-Crtl1	Link protein-1/WB, IHC	Goat monoclonal	1:1000	R&D Systems
Anti-NF200	Neural filaments 200 kDa/IHC	Mouse monoclonal	1:200	Sigma-Aldrich, St. Louis, MO, USA
SMI-32	Nonphosphorylated neurofilament H/IHC	Mouse monoclonal	1:1000	Covance, Princeton, NJ, USA
Anti-NeuN	Neural DNA/IHC	Rabbit monoclonal	1:200	Millipore
Anti-MTC02	Non-glycosylated protein component of human mitochondria/IHC	Mouse monoclonal	1:200	Abcam
Anti-β-III tubulin	β-tubulin isotype III/IHC	Mouse monoclonal		Sigma-Aldrich
Anti-S100β	β chain/IHC/ICC	Rabbit polyclonal	1:400	DAKO Denmark, Glostrup, Denmark
Anti-l-type Ca^2+^ CP α1C	L-type Ca^2+^ CP α1C/ICC	Rabbit polyclonal	1:200	Alomone Labs, Jerusalem, Israel
Anti-N type Ca^2+^ CP α1B	N type Ca^2+^ CP α1B/ICC	Rabbit polyclonal	1:200	Alomone Labs
Anti-P/Q-type Ca^2+^ CP α1A	P/Q-type Ca^2+^ CP α1A/ICC	Rabbit polyclonal	1:200	Alomone Labs
Anti-Ryanodine Receptor 1	Ryanodine Receptor 1/ICC	Rabbit polyclonal	1:200	Alomone Labs
Anti-Ryanodine Receptor 2	Ryanodine Receptor 2/ICC	Rabbit polyclonal	1:200	Alomone Labs
Anti-Ryanodine Receptor 3	Ryanodine Receptor 3/ICC	Rabbit polyclonal	1:100	Alomone Labs
Anti-ORAI Receptor 1	ORAI Receptor 1/ICC	Rabbit polyclonal	1:200	Alomone Labs
Anti-ORAI Receptor 2	ORAI Receptor 2/ICC	Rabbit polyclonal	1:200	Alomone Labs
Anti-ORAI Receptor 3	ORAI Receptor 3/ICC	Rabbit polyclonal	1:1000	Sigma-Aldrich
Anti-Hepcidin	Hepcidin	Goat monoclonal	1:200	Dr. Raha-Chowdhury
Anti-HB9	HB9 homeobox transcription factor	Goat polyclonal	1:200	Santa Cruz Inc., Dallas, TX, USA
Anti-SR	Serotonin	Goat polyclonal	1:600	Abcam
Anti-ParAlb	PV 25 Rabbit anti Parvalbumin	Rabbit	1.2000	Swant, Marly, Switzerland
NKx6.1	NK homeodomain protein, Nkx6.1	Mouse monoclonal	1:20	DSHB
Anti-Islet1	Islet 1- Neural Stem Cell Marker	Rabbit polyclonal	1:500	Abcam
Anti-Islet2	GST fusion from E. coli linker region of protein	Mouse	1:20	DSHB
Anti-CHAT	Choline Acetyltransferase	Chicken polyclonal	1:50	Abcam
Anti-Nestin	Anti-NESTIN	Mouse monoclonal	1:2500	Millipore,
Anti-GFAP	Anti-glial fibrillary acidic protein	Mouse monoclonal	1:1000	Cell Signaling, Danvers, MA, USA

**Table A4:** Gene expression assays.

Assay	Gene Name
Rn00573424_m1	*aggrecan*
Rn00581331_m1	*neurocan*
Rn00563814_m1	*brevican*
Rn00564869_m1	*tenascin-R*
Rn01493755_m1	*versican*
Rn00569884_m1	*hapln1*
Rn99999125_m1	*bcl2*
Rn00563902_m1	*casp3*
Rn00573917_m1	*casp7*
Rn01480161_g1	*bax*
Rn01533872_m1	*ngf*
Rn02531967_s1	*bdnf*
Rn 00710306_m1	*igf-1*
Rn 00580526_m1	*ncam*
