# Supplementary material for: Transplantation of Neural Precursors Derived from Induced Pluripotent Cells Preserve Perineuronal Nets and Stimulate Neural Plasticity in ALS Rats
Source: Int J Mol Sci. 2020 Dec 16;21(24):9593. doi: 10.3390/ijms21249593 (PMC7766921; doi:10.3390/ijms21249593)
Supplement: Supplementary file 1 [file ijms-21-09593-s001.pdf]

Supplementary Material

Transplantation of Neural Precursors Derived from Induced Pluripotent Cells Preserve Perineuronal Nets and Stimulate Neural Plasticity in ALS Rats

2. Results

2.1. Differentiation and Maturation of NP-iPS in Culture

Culturing of NP-iPS for 4 weeks in the neural differentiation medium following Hu's protocol [1] stimulated NP-iPS differentiation toward neural cells with neuronal phenotype (Figure S1). Our NP-iPS cultures formed networks and bundles of cells, which also contained cells stained for typical motoneuronal (CHAT, Islet1, HB9), but also neural (Nkx6.1, NF200, SMI32 and Nestin) and astrocytic (S100β, GFAP) markers (Figure S1A-F). We studied action potentials of randomly selected cells of various morphology during of differentiation by patch-clamp (Figure S2). We measured a total number of 39 cells and identified six groups of cells (Figure S2 A, B, C, D, E, F) according to their passive (V_m, IR, C_m) and active (K_{DR}, K_{IR}, K_A, and Na^+ current components, AP) current patterns and electrophysiological properties (for more information, see supplementary Table S1). Our electrophysiological data identified electrophysiological patterns of cells typical for: a) oligodendroglia (Figure S2A (DEC; 12.8% of the total number of cells); Figure S2B, DEC+OUT; 5.1% of the total number of cells) and Figure S2C (OUT+T; 10.3% of the total number of cells); b) neuronal precursors (Figure S2D1, OUT; 25.6% of the total number of cells) and cells with the Na^+ currents (Figure S2D2a; 40.0% of OUT) and action potentials (Figure S2D2b; 20.0% of OUT); c) astrocytes (Figure S2E, PAS; 30.8% of the total number of cells); d) unknown cells with a very small voltage-activated K^+ currents (Figure S2F no current; 15.4% of the total number of cells).

Supplementary Table 1. Electrophysiological properties of NP-iPS cells that were used for transplantation.

Current		Vm [mV]	IR [MΩ]	Cm [pF]	KIR [pA]	[pA/pF]	KDR [pA]	[pA/pF]	KA [pA]	[pA/pF]	Na [pA]	[pA/pF]
DEC	Mean	-80.6	82.69184522	36.52	176.2	5.601783983	426.32	13.87605686	-	-	-	-
	SEM	1.3446189	9.421514021	6.202863855	25.27215068	1.530795805	30.81372941	3.292004488	-	-	-	-
DEC+OUT	Mean	-83	82.09201695	114	372.25	2.950781055	826.9	6.890469464	-	-	-	-
	SEM	0	23.88807398	21.92031022	157.9322996	0.817984767	258.6596606	0.944021337	-	-	-	-
no current	Mean	-50.8	1103.462046	14.34	43.7	3.1203644	83.62	5.708722421	-	-	-	-
	SEM	5.2869651	390.5550697	2.503693272	7.701116802	0.528294145	17.16035897	0.158900973	-	-	-	-
OUT	Mean	-51.875	1224.326112	15.375	78.4	4.569294934	521.675	48.60015407	423.3	44.55789	432.675	45.2729
	SEM	5.3222337	358.7925567	3.264187782	21.69525038	1.134557117	102.0916925	14.10531973	0	0	225.0327	23.63349
OUT+T	Mean	-57.25	813.0674993	30.4	217.05	8.380167852	1023.2	32.83804326	-	-	-	-
	SEM	9.9144780	380.532052	6.296328295	51.39861987	2.568746513	264.8502478	2.951453632	-	-	-	-
PAS	Mean	-69.333333	67.30610339	34.48333333	87.45	2.478128768	261.025	8.501857274	-	-	-	-
	SEM	1.7132382	8.931365318	6.042486534	21.74609045	0.501423862	52.28513322	1.649378276	-	-	-	-

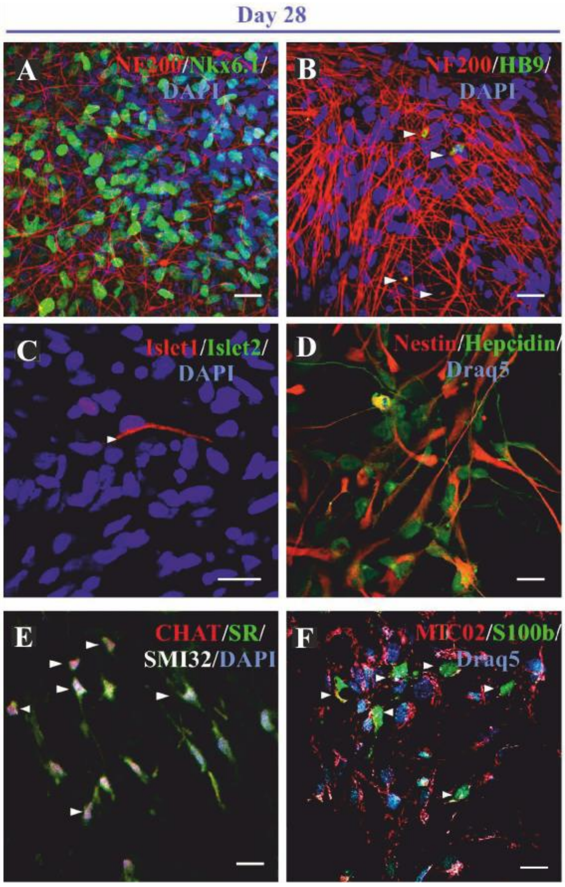

Figure S1. NP-iPS characteristics during neuronal differentiation and maturation. At the final stage of differentiation (Day 28, **A-F**) cells formed bundles from NF200-positive neurites, Nkx6.1-positive (**A**), HB-9-positive (**B**) and Islet1-positive (**C**) neurons and islet2-negative cells (**C**). All nestin-positive and nestin-negative cells expressed hepcidin (**D**). At the 28th day of differentiation we had a mixed cell cultures containing CHAT-, SMI32- and SR-positive neurons (**E**) and S100β-positive astrocytes (**F**). Cell nuclei were visualized either with DAPI or DRAQ5 staining. Scale bars = 20μm.

2.2. Differentiation of NP-iPS is Accompanied with Differential Expression of Ca^{2+} Channels

Having established that the NP-iPS cells could differentiate, we asked whether the various cell types in the cultures show a characteristic pattern of channel expression. We found that NP-iPS derived neurons were also electrically active and showed spontaneous calcium oscillations ($(Ca^{2+})_i$). These oscillations are closely similar to those observed in the dissociated cultures of purified embryonic (E15) rat motoneurons [2].

The functional properties of NP-iPS were studied during the neuronal differentiation following Hu's protocol, starting from day 7 in vitro (week 1, mixed culture of neural cells), each 7 days, up to day 28 (week 4, mixed culture of mature glial and neuronal cells). The ICC analysis of $(Ca^{2+})_i$-sensitive channels and receptors was performed simultaneously with $(Ca^{2+})_i$ measurements (Figure S3A-F). No significant differences in the channel expression or function were observed between week 3 and 4 of differentiation, therefore in this study only data obtained during weeks 1–3 is presented. The resting level of $(Ca^{2+})_i$ in NP-iPS during the first two weeks of differentiation remained stable being 56 ± 0.01 nM (n=58), 54 ± 0.01 nM (n=61) during the first and second week respectively, and increased significantly to 80 ± 0.01 nM (n=49) during the third week of differentiation.

The voltage-gated Ca^{2+} channels (VGCC), glutamate and purinergic receptors as well as Ca^{2+} release from the intracellular stores play an important role in the function and survival of MNs. The changes in $(Ca^{2+})_i$ were monitored in response to 50 mM K^+ depolarizations, 100 μM glutamate, 100

µM kainic acid (KA), 100 µM *n*-methyl- D-aspartic acid (NMDA), 100 µM ATP (all from Sigma-Aldrich; St. Louis, MO, USA), 10 µM cyclopiasonic acid (CPA; Alomone Labs Ltd., Jerusalem, Israel). Throughout the whole duration of differentiation all tested cells were sensitive to CPA, K^+, glutamate, KA, NMDA and ATP, suggesting the functional intracellular $(Ca^{2+})_i$ stores, Ca^{2+} channels, glutamate and purinergic receptors (Figure S3A). During week 3 of differentiation 88% (n = 16) of NP-iPS responded with the rise of $(Ca^{2+})_i$ to the application of 100 µM glutamate. Among glutamate-sensitive cells 25% of cells tested were also sensitive to 100 µM NMDA and all cells were also sensitive to 100 µM KA. A small subpopulation (about 12%) of NP-iPS starting from week 2 of differentiation exhibited spontaneous $(Ca^{2+})_i$ transients in the absence of any stimuli (Figure S3B). The frequency of these spontaneous $(Ca^{2+})_i$ transients increased significantly (p = 0.02) during the third week of differentiation and remained stable onwards, being 11.6 ± 0.001 mHz at week 2 and 23.7 ± 0.003 mHz at week 3.

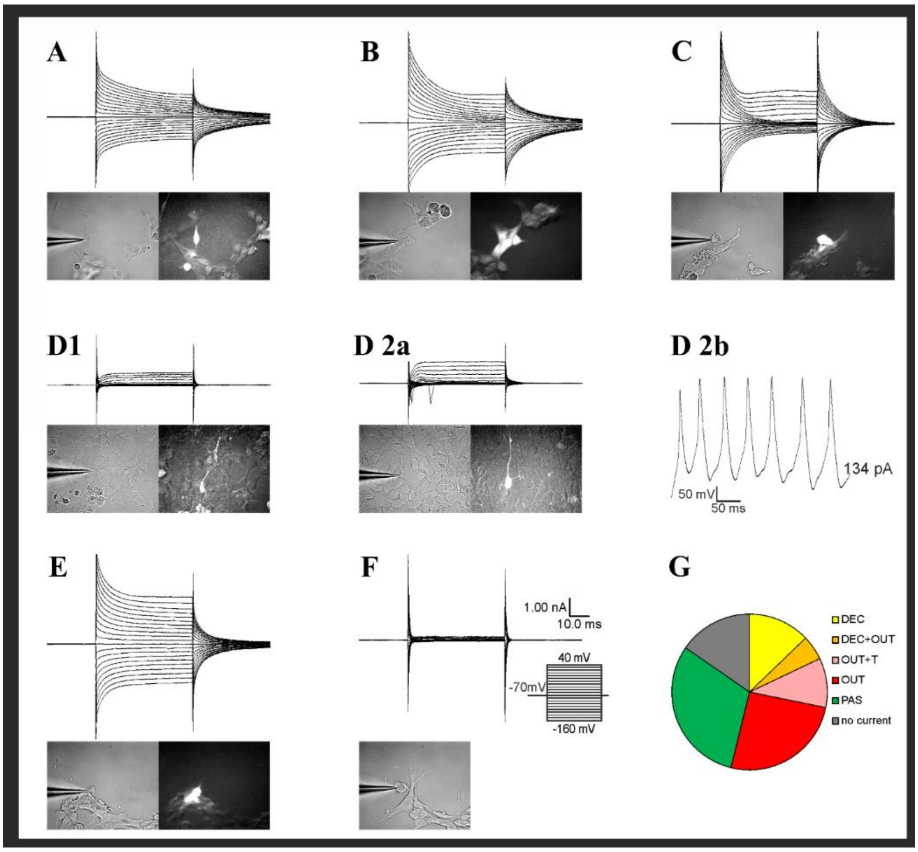

Figure S2. Patch clamp current measurements from NP-iPS cells at the stage of neural precursors. NP-iPS cells from three different cultures (n=39) were randomly selected and measured for electrophysiological properties. Depending on the passive (V_m, IR, C_m) and active current patterns (K_{DR}, K_{IR}, K_A, and Na^+ current components, AP) cells were divided into 6 different groups with different electrophysiological properties (A, B, C, D, E, F; for details please refer to supplement Table 1). (**A**) show cells displaying symmetrical, passive K^+ currents, decaying during the duration of the voltage pulse, with tail currents, which is typical for oligodendrocytes (DEC; 12.8% of the total number of cells). (**B**) demonstrates cell with a similar current profile to that of DEC, but with higher amplitudes of K_{DR} (DEC+OUT; 5.1% of the total number of cells). (**C**) represents a typical cell with high current densities of K_{DR}, accompanied with tail currents (OUT+T; 10.3% of the total number of cells). (**D1**) shows cell with high current densities of K_{DR} and K_A, which is typical of neuronal precursors (OUT; 25.6% of the total number of cells). Some of the cells showed Na^+ currents (**D2a**; 40.0% of OUT) and action potentials (**D2b**; 20.0% of OUT). (**E**): These cells showed a passive current pattern, predominantly time- and voltage-independent non-decaying K^+ currents, with only small voltage-activated K^+ currents. This current pattern is typical of astrocytes (PAS; 30.8% of the total number of cells). (**F**): This cell type expressed only very small voltage-activated K^+ currents (no

current; 15.4% of the total number of cells). (**G**): Diagram showing the incidence of the cell types identified in (**A-F**). Magnification 400 ×.

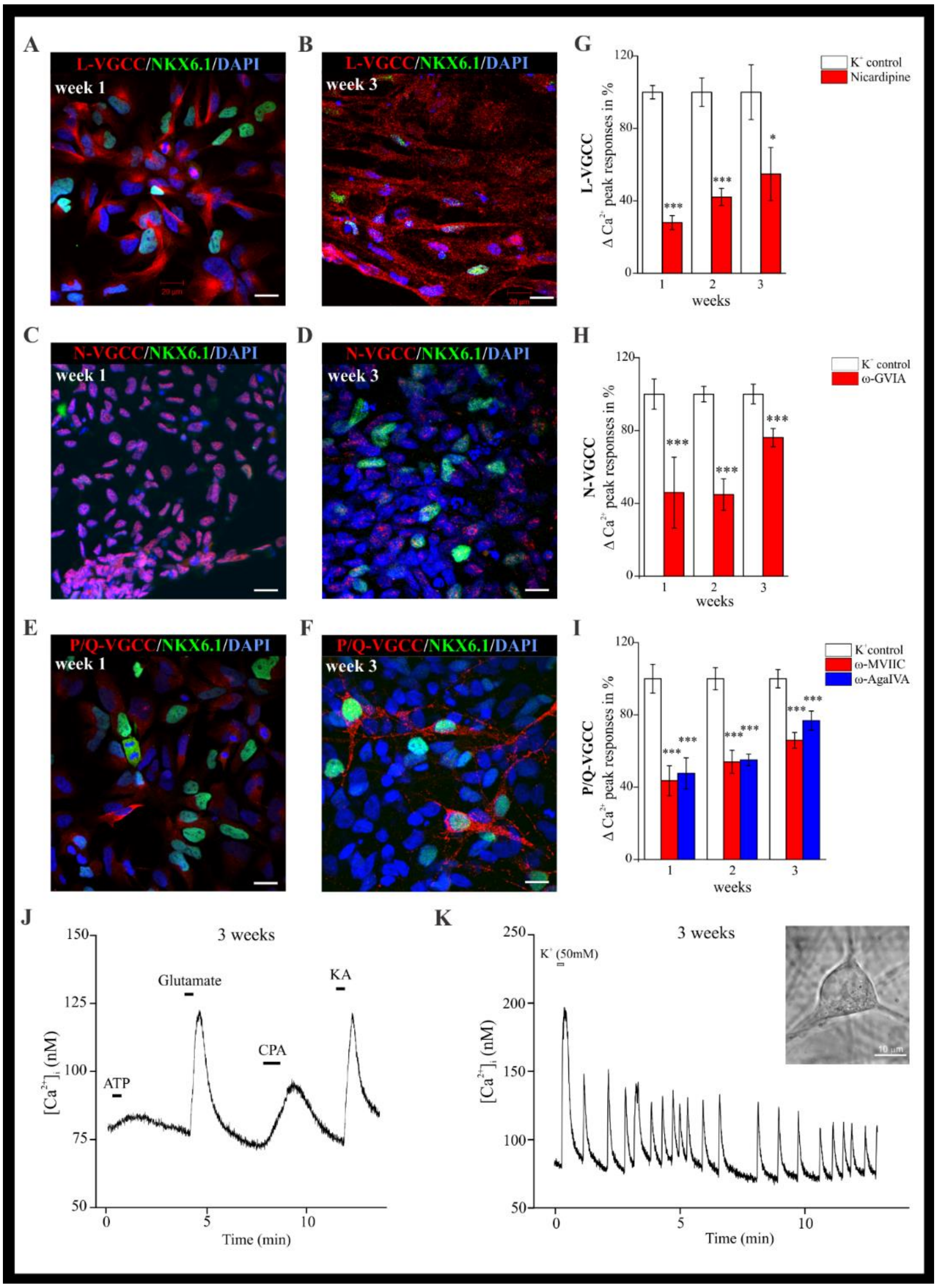

Figure S3. Functional properties of NP-iPS-derived motor neuron-like cells. The expression and function of various Ca^{2+}-sensitive channels and receptors were analyzed in NP-iPS differentiated to motoneurons every week starting from day 7, using intracellular $(Ca^{2+})_i$ measurements and immunocytochemical analysis. A representative trace of the Ca^{2+} measurements shows the typical $(Ca^{2+})_i$ increase in response to various physiological stimuli: ATP (100 µM), glutamate (100 µM), kainic acid (KA; 100 µM) and cyclopiasonic acid (CPA; 10 µM). Spontaneous $(Ca^{2+})_i$ oscillations could be observed in a subpopulation of NP-iPS-derived MNs starting from the second week of differentiation **(A)**. The representative trace showing 50mM K^+-induced $(Ca^{2+})_i$ increase and spontaneous oscillations observed in a NP-iPS-derived MNs after 3 weeks of differentiation **(B)**. L,-N- and *p*/Q-type of voltage-gated Ca^{2+} channels (VGCC) were expressed and functional during the differentiation of NP-iPS to motoneurons **(C-K)**. The bar diagrams represent the cumulative values (evoked peak amplitude, mean ± SEM) obtained from 10 to 62 cells in each of the experimental conditions, expressed as the

percentage of K^+-evoked $(Ca^{2+})_i$ responses in the absence (control) and presence of different VGCC blockers: 1μM nicardipine (for L-type) (**C**); 800nM ω-conotoxin GVIA (for N-type) (**F**); 300nM ω-conotoxin MVIIC and 300nM ω-agatoxin IVA (for P/Q-type) (**I**). Peak amplitude of K^+-induced $(Ca^{2+})_i$ responses was taken as 100% (control). **p* < 0.05; ****p* < 0.001. Confocal images showing the co-localization of immunocytochemical staining for motor neuronal marker Nkx6.1 (green) and various subunits of VGCC (red): α_{1c} for L-type **(D, E)**,α_{1b} for N-type **(G, H)**, and α_{1a} for P/Q-type **(J, K)** after 1 week **(D, G, J)** and 3 weeks **(E, H, K)** of neuronal differentiation. Cell nuclei were visualized with DAPI staining. Scale bars = 20 μm.

To study the Ca^{2+} entry through the VGCC the $(Ca^{2+})_i$ transients were evoked by depolarization by 50 mM K^+. The application of high K^+ solution elicited a rapid increase in $(Ca^{2+})_i$ in 45–78% of tested cells throughout the whole period of differentiation. To characterize the specific involvement of various subtypes of VGCC, we used specific Ca^{2+} channel blockers (all purchased from Alomone Labs Ltd., Jerusalem, Israel) such as nicardipine (for L-type), ω-conotoxin GVIA (for N-type), ω-conotoxin MVIIC and ω-agatoxin IVA (for P/Q-type). Preincubation of cells with 1 μM nicardipine significantly inhibited K^+-induced $(Ca^{2+})_i$ increase by 72 ± 3.7% ($p < 0.001$, $n = 40$) during week 1, by 58 ± 4.7% ($p <$ 0.001; $n = 19$) during week 2 and by 45 ± 14.7% ($p = 0.02$; $n = 10$) during week 3 of differentiation (Figure S3C). The immunocytochemical staining for the α_{1C} subunit of Ca^{2+} channels confirmed the expression of L-VGCC in iPS-derived neurons (Figure S3D,E) A specific N-type channel blocker ω-conotoxin GVIA at 800 nM concentration effectively inhibited K^+-induced $(Ca^{2+})_{i\ i}$ increase throughout the whole period of differentiation, suggesting the expression of functional N-type Ca^{2+} channels. The inhibition caused by GVIA was by 54 ± 19.4% ($p < 0.001$, $n = 7$) during week one, by 55 ± 8.6% ($p <$ 0.001; $n = 48$) during week two and by 24 ± 5% ($p < 0.001$; $n = 36$) during week three (Figure S3F). The presence of N-type VGCC (α_{1B}) was also confirmed by immunocytochemistry (Figure S3G,H). Specific P/Q-type blockers, ω-conotoxin MVIIC (ω-MVIIC) or ω-agatoxin IVA (ω-Aga IVA), each applied at 300 nM each, significantly decreased the K^+-induced $(Ca^{2+})_i$ rise in iPS-derived neurons (Figure S3I). The inhibition caused by ω-MVIIC was by 57 ± 8.3% ($p < 0.001$; $n = 38$) during week 1, by 46 ± 6.4% ($p < 0.001$; $n = 21$) during week 2 and by 34 ± 4.3 % ($p < 0.001$; $n = 37$) during week 3. ω-Aga IVA in its turn inhibited K^+-induced $(Ca^{2+})_i$ transients by 52 ± 8.6% ($p < 0.001$; $n = 35$) during week 1, by 45 ± 3.2% ($p < 0.001$; $n = 62$) during week 2 and by 23 ± 5.3% ($p < 0.001$; $n = 48$) during week 3 (Figure S3I). In addition, these cells also exhibited $(Ca^{2+})_i$ responses (traces obtained from an example is shown in Figure S3J) to ATP (100 μM), glutamate (100 μM), cyclopiasonic acid (CPA; 10 μM) and kainic acid (KA; 100 μM). The majority of NP-iPS during the first week of differentiation expressed α_{1A} subunit (P/Q-LVCC) on immunocytochemistry. Interestingly, after three weeks of differentiation the immunostaining for P/Q-VGCC was localized only in Nkx6.1$^+$ cells (Figure S3K), while 4L- and N-VGCC were expressed both by Nkx6.1$^+$ and Nkx6.1$^-$ cells (Figure S3E,H). Overall, these results show a progressive development of the ion channel profile of the cells consistent with neuronal differentiation.

4. Materials and Methods

4.1. Differentiation of NP-iPS Toward Neuronal Phenotype

The differentiation of NP-iPS toward more mature neurons was reached by adapting the protocol described by Hu and Zhang for the differentiation of embryonic stem cells [1]. After thawing, NPs-iPS were cultured in enriched DMEM/F12-Neurobasal media (described above) to get rid of dead cells debris and to establish a monolayer culture. Starting from the seventh day (which corresponds to step 42 of Hu protocol) NPs-iPS were cultured in the Neural differentiation medium (DMEM/F12/N2; heparin; cAMP, 1:10,000; ascorbic acid, 1:1,000; BDNF, 1:10,000; GDNF, 1:10,000 and IGF-1, 1:10,000) that initiated advanced neuronal differentiation. Fourteen days after neuronal differentiation began, cells were allowed to mature for another two weeks in neural differentiation medium (Figure S1). In parallel to the aforementioned experiments, some NP-iPS were plated on laminin- and and poly-L-ornithine-coated (Sigma-Aldrich, St. Louis, MO, USA) 22mm glass-bottom dishes (WillCo Dishes BV, Amsterdam, Netherlands) and underwent the same differentiation

protocol. These cell-cultures at every stage of differentiation (i.e., every seventh day) were used for intracellular Ca^{2+} measurements $(Ca^{2+})_i$ and/or immunocytological (ICC) staining.

4.2. Measurements of $(Ca^{2+})_i$

The $(Ca^{2+})_i$ measurements were performed according to previously reported methods [3,4]. Briefly, the cells were plated on 22 mm glass-bottom dishes coated with laminin and poly-L-ornithine (both from Sigma-Aldrich, St. Louis, MO, USA), were incubated with 2.5 µM Fura-2 AM (Invitrogen, Carlsbad, CA, USA) and 0.02% Pluronic F-127 (Molecular Probes, Eugene, OR, USA) in culture medium at 37 °C and 5% CO_2 for 40 min. Loaded cells were then washed and the culture medium replaced with Normal Locke's buffer containing (in mM): NaCl, 140; KCl, 5; $MgCl_2$, 1.2; $CaCl_2$, 2.2; glucose, 10; HEPES-Tris, 10; pH 7.25, osmolarity 298–300 mosmol/L^{-1}) and kept at 37 °C throughout the time course of the experiment. Fluorescence measurements of $[Ca^{2+}]_i$ were performed either using a fluorescence microspectrofluorimetry system (FFP; Fast Fluorescence Photometer, Zeiss, Jena, Germany) for single-detector experiments [3,4] or using a CCD video-imaging system based on an inverted microscope AxioObserver D1 (Zeiss) equipped with a CCD camera and Lambda-DG4 fast rotating wheel illumination system (Sutter Instrument, Novato, USA) for double excitation at 340 and 380 nM, for $[Ca^{2+}]_i$ measurements on multiple cells [5]. To estimate $(Ca^{2+})_i$ in nM a calibration was performed on a few cells for both systems. An estimation of $(Ca^{2+})_i$ was determined from the f340/f380 ratio using the Grynkiewicz equation [6]. The calibration parameters for the FFP system were R_{min} = 0.20, R_{max} 3.16, β = 2.86. The calibration performed with the imaging system gave R_{min} = 0.2, R_{max} = 7.2, β = 7.7. The dissociation constant for Fura-2 at 37°C was assumed as K_D = 224 nM. The control and test solutions were exchanged using a multiple capillary perfusion system, as described previously [2], with appropriate modifications [7] using a computer controlled multichannel peristaltic pump (REGLO ICC, Ismatec, Germany). Concentrated stock solutions of nicardipine and glutamate were prepared in DMSO, while the remaining stock solutions of agonists/antagonists were dissolved in dH_2O. All concentrated stock solutions were stored at −20 °C. Test solutions were prepared daily using aliquots from frozen stocks to obtain the working concentrations.

4.3. Data Analysis and Statistical Methods

To analyze the calcium measurement data, Origin 8.5.1 was employed for plotting and statistical procedures. The results are expressed as mean ± SEM. The sample size (N) given is the number of cells tested according to the same protocol (control, test drug, recovery) for each group. One-way ANOVA for multiple comparisons were used to determine significant differences between the experimental groups. Values of $*p \leq 0.05$ and $**p \leq 0.01$, and $***p \leq 0.001$ were considered significant.

4.4. Patch-Clamp Recording

Membrane currents were recorded using a patch-clamp technique in the whole-cell configuration. Recording pipettes with a tip resistance of ~10 MΩ were made from borosilicate capillaries (Sutter Instruments, Novato, CA, USA), using a P-97 Brown-Flaming micropipette puller (Sutter Instruments, Novato, CA, USA). Recording pipettes were filled with intracellular solution containing (mM): 130 KCl, 0.5 $CaCl_2$, 2 $MgCl_2$, 5 EGTA, 3 ATP, 10 HEPES (pH 7.2) and additionally Alexa Fluor hydrazide 488 (A488; Molecular Probes, Carlsbad, CA, USA) to visualize recorded cells. Those cells labeled with AF hydrazide were further used for post-recording immunocytochemical identification. All recordings were made in artificial cerebrospinal fluid (aCSF), containing (mM): 135 NaCl, 2.7 KCl, 2.5 $CaCl_2$, 1 $MgCl_2$, 1 Na_2HPO_4, 10 glucose, and 10 HEPES (osmolality 312.5 ± 2.5 mmol/kg, pH 7.4). The recordings were made on coverslips perfused with aCSF at 22°C. Electrophysiological data were measured with a 10 kHz sample frequency, using an EPC9 amplifier, controlled by PatchMaster software (HEKA Elektronik, Lambrecht/Pfalz, Germany), and filtered using a Bessel filter. The coverslips with cells were transferred to the recording chamber of an upright Axioscop microscope (Carl Zeiss, Jena, Germany), equipped with electronic micromanipulators (Luigs & Neumann, Ratingen, Germany) and a high-resolution AxioCam HR digital camera (Carl

Zeiss, Jena, Germany). The resting membrane potential (V_m) was measured by switching the EPC9 amplifier to the current-clamp mode. Using FitMaster software (HEKA Elektronik, Lambrecht/Pfalz, Germany), membrane resistance (IR) was calculated from the current value of 40 ms, after the onset of the depolarizing 10 mV pulse from the holding potential, of −70 mV to −60 mV for 50 ms. Membrane capacitance (C_m) was determined automatically from the Lock-in protocol by PatchMaster. Current patterns were obtained by hyper-, and by depolarizing the cell membrane from the holding potential of −70 mV to the values ranging from −160 mV to 40 mV, at 10 mV intervals. Pulse duration was 50 ms. In order to isolate the delayed outwardly rectifying K^+ current (K_{DR}) current components, a voltage step from −70 to −60 mV was used to subtract the time- and voltage-independent currents, as described previously [8] To activate the K_{DR} currents only, the cells were held at −50 mV, and the amplitude of the K_{DR} currents was measured at 40 mV, at the end of the pulse. The inwardly rectifying K^+ (K_{IR}) currents were determined at −160 mV, at the end of the pulse. The fast activating and inactivating outwardly rectifying K^+ (K_A) currents were isolated by subtracting the current traces, clamped at −110 mV from those clamped at −50 mV, and its amplitude was measured at the peak value. Depolarizing steps from −70 to 20 mV activated Na^+ currents and current component was isolated by subtraction of the current traces clamped at the voltage with maximal current activation, and its amplitude was measured at the peak value. Current densities were calculated by dividing the maximum current amplitudes by the corresponding C_m values, for each individual cell. Sodium current amplitudes were measured at the peak value. Action potentials (AP) were obtained in the current-clamp mode. Current values ranged from 50 pA to 1 nA, at 50 pA intervals. Pulse duration was 300 ms. After recording, the coverslips were fixed in phosphate buffer (PB; 0.2 M; pH 7.4), containing 4% paraformaldehyde for 9 min, and then transferred to phosphate-buffered saline (PBS; 10 mM; pH 7.2), for post-recording identification using immunocytochemistry.

References

1. Hu, B.Y.; Zhang, S.C. Differentiation of spinal motor neurons from pluripotent human stem cells. *Nature protocols* **2009**, *4*, 1295–1304.
2. Dayanithi, G.; Mechaly, I.; Viero, C.; Aptel, H.; Alphandery, S.; Puech, S.; Bancel, F.; Valmier, J. Intracellular ca2+ regulation in rat motoneurons during development. *Cell calcium* **2006**, *39*, 237–246.
3. Forostyak, O.; Romanyuk, N.; Verkhratsky, A.; Sykova, E.; Dayanithi, G. Plasticity of calcium signaling cascades in human embryonic stem cell-derived neural precursors. *Stem Cells Dev.* **2013**, *22*, 1506–1521.
4. Forostyak, O.; Butenko, O.; Anderova, M.; Forostyak, S.; Sykova, E.; Verkhratsky, A.; Dayanithi, G. Specific profiles of ion channels and ionotropic receptors define adipose- and bone marrow derived stromal cells. *Stem cell research* **2016**, *16*, 622–634.
5. Kortus, S.; Srinivasan, C.; Forostyak, O.; Ueta, Y.; Sykova, E.; Chvatal, A.; Zapotocky, M.; Verkhratsky, A.; Dayanithi, G. Physiology of spontaneous $[ca^{(2+)}]_i$ oscillations in the isolated vasopressin and oxytocin neurones of the rat supraoptic nucleus. *Cell calcium* **2016**, *59*, 280–288.
6. Grynkiewicz, G.; Poenie, M.; Tsien, R.Y. A new generation of ca^{2+} indicators with greatly improved fluorescence properties. *Journal of Biological Chemistry* **1985**, *260*, 3440–3450.
7. Kortus, S.; Srinivasan, C.; Forostyak, O.; Ueta, Y.; Sykova, E.; Chvatal, A.; Zapotocky, M.; Verkhratsky, A.; Dayanithi, G. Physiology of spontaneous [ca] oscillations in the isolated vasopressin and oxytocin neurones of the rat supraoptic nucleus. *Cell calcium* **2016**, *59*, 280–288.
8. Anderova, M.; Kubinova, S.; Jelitai, M.; Neprasova, H.; Glogarova, K.; Prajerova, I.; Urdzikova, L.; Chvatal, A.; Sykova, E. Transplantation of embryonic neuroectodermal progenitor cells into the site of a photochemical lesion: Immunohistochemical and electrophysiological analysis. *Journal of neurobiology* **2006**, *66*, 1084–1100.

Publisher's Note: MDPI stays neutral with regard to jurisdictional claims in published maps and institutional affiliations.

© 2020 by the author. Licensee MDPI, Basel, Switzerland. This article is an open access article distributed under the terms and conditions of the Creative Commons Attribution (CC BY) license (http://creativecommons.org/licenses/by/4.0/).
